# Quality measurement for cardiovascular diseases and cancer in hospital value-based healthcare: a systematic review of the literature

**DOI:** 10.1186/s12913-022-08347-x

**Published:** 2022-08-01

**Authors:** Rawia Abdalla, Milena Pavlova, Mohammed Hussein, Wim Groot

**Affiliations:** 1 Department of Health Services Research, CAPHRI, Maastricht University, Maastricht University Medical Center, Faculty of Health, Medicine and Life Sciences, Maastricht, Limburg The Netherlands; 2 Department of Hospitals Accreditation, Saudi Central Board for Accreditation of Healthcare Institutions (CBAHI), Riyadh, Saudi Arabia; 3 Maastricht University, Top Institute Evidence-Based Education Research (TIER), Maastricht, Limburg The Netherlands

**Keywords:** Quality measurement, Value-based healthcare, Hospital, Cardiovascular, Cancer

## Abstract

**Background:**

This systematic literature review identifies hospital value-based healthcare quality measures, measurement practices, and tools, as well as potential strategies for improving cardiovascular diseases and cancer care.

**Methods:**

A systematic search was carried out in the PubMed, Embase, CINAHL, and MEDLINE (OvidSP) databases. We included studies on quality measures in hospital value-based healthcare for cardiovascular diseases and cancer. Two reviewers independently screened titles and abstracts, conducted a full-text review of potentially relevant articles, assessed the quality of included studies, and extracted data thematically. This review was conducted in accordance with the Preferred Reporting Items for Systematic Reviews and Meta-Analyses guidelines, and four validated tools were used for methodological quality assessment.

**Results:**

The search yielded 2860 publications. After screening the titles and abstracts, 60 articles were retrieved for full-text review. A total of 37 studies met our inclusion criteria. We found that standardized outcome sets with patient involvement were developed for some cardiovascular diseases and cancer. Despite the heterogeneity in outcome measures, there was consensus to include clinical outcomes on survival rate and disease control, disutility of care, and patient-reported outcome measures such as long-term quality of life.

**Conclusion:**

Hospitals that developed value-based healthcare or are planning to do so can choose whether they prefer to implement the standardized outcomes step-by-step, collect additional measures, or develop their own set of measures. However, they need to ensure that their performance can be consistently compared to that of their peers and that they measure what prioritizes and maximizes value for their patients.

**Trial registration:**

PROSPERO ID: CRD42021229763.

**Supplementary Information:**

The online version contains supplementary material available at 10.1186/s12913-022-08347-x.

## Background

Value-Based Healthcare (VBHC) is an approach to improving healthcare systems that attracts increasing attention around the world. One commonly used definition of value is the best health outcomes at the lowest cost [1]. Frequently, VBHC considers improving the health outcomes that are most relevant to patients. This is seen as an essential goal to enhance patient experience and optimize services [2–4]. The number of VBHC programmes is growing rapidly, which necessitates establishing definitions and measurements of value and aligning healthcare services with VBHC concepts [2–4]. Rigorous measurement of value and health outcomes should be at the center of these initiatives [5].

In a VBHC context, it is important to understand that measures of quality can refer to the structure, process, and outcome components of the healthcare provision [6]. These outcomes include clinical outcomes reported by healthcare providers as well as Patient-Reported Outcomes Measures (PROMs) and Patient-Reported Experience Measures (PREMs) [7]. Patient-reported measures have an important role in VBHC as they capture the category of outcomes reported by patients that matter most to them, such as pain, anxiety, and physical status. Integrating them into outcome measurement ensures that the focus is shifted not only to outcome data reported by clinicians but also to outcomes experienced by the patients. For instance, an improved ejection fraction for a patient with heart failure is a clinical outcome while quality of life is an important PROM that captures other measures such as functional status. Ideally, these quality measures must be measured over the entire care cycle of any medical condition that includes prevention, diagnosis, treatment, rehabilitation, patient engagement, follow-up, and procurement [8]. To align these key quality measurement models with VBHC, it is critical to operationalize the theoretical concept of value into quality measures using standardized, well-defined, and risk-adjusted measure sets that are relevant to the patient. These measures can be further analyzed to indicate whether services are of good or poor quality and to facilitate global comparisons and shared learning of best practices [9].

Thus, initiatives have been developed around the world, and various sets of outcomes have been proposed along with case-mix variables for multiple medical conditions [3]. The International Consortium for Healthcare Outcome Measurement (ICHOM), a nonprofit organization founded in 2012 by Harvard Business School professor Michael Porter, is one of the leading organizations in this realm with the ultimate goal of defining globally standardized sets of outcomes that matter most to patients as well as developing measurement tools and time points to facilitate comparisons across healthcare systems [10]. Other initiatives have been established and aligned with VBHC tenets such as the VBHC Center Europe, the Organization of Economic Co-operation and Development (OECD), the Care Institute in the Netherlands, the Value-Based Health Care Delivery (VBHCD), and the National Health Service (NHS) England [11–15]. Despite this advancement in outcome measurement, the transformation toward VBHC is not without obstacles. The lack of consensus on standardized outcome measures for many medical conditions [10], the debate regarding the use of administrative data (e.g., claims) versus obtaining meaningful clinical data [16, 17], the argument over the validity and reliability of process measures in VBHC models and whether a causal process-outcome relationship exists [1, 18, 19], and the controversial approach in selecting, collecting, and reporting relevant PROMs measures [20], have all been highlighted as challenges.

These challenges have been reported in the quality measurement of different medical conditions such as cardiovascular disease and cancer, which are considered the first and second most common causes of death globally, respectively [21]. In addition to the high morbidity and mortality burden of these two medical conditions, they are costly diseases for healthcare systems and society. Moreover, the cardiovascular and cancer communities have developed multiple quality measures of compliance and performance (e.g., heart failure). These measures have been used in quality improvement, value-based payment models, reimbursement, accreditation, and public reporting [4, 17], and several healthcare systems would prioritize improving outcomes in these conditions. Accordingly, these conditions have a well-established quality measurement practice and as they include a large number of diseases and conditions, reviewing them may enhance the opportunity to identify the potential variety of quality measurement practices across different medical conditions. This could be not possible if we focus on one area of conditions as each disease has its specific outcomes. At the same time, it is unfeasible to review all medical conditions in a single research.

Therefore, the research question of this review is as follows: what cardiovascular and oncology outcome measures are actually used or recommended in hospital VBHC initiatives? The aim is to identify the used and recommended hospital VBHC quality measures, measurement practices, and tools, and to identify potential strategies for improving cardiovascular diseases and cancer care in order to provide evidence for hospital management systems on the recommended VBHC quality measurement practices. No similar reviews were found as the published literature on this topic reviewed quality measures for various medical conditions but did not focus on cardiovascular or cancer diseases [22–26], or did not focus on the VBHC context [22, 27–30].

## Methodology

We carried out a systematic literature review on the quality measures used in hospitals to deliver value-based cardiovascular and cancer care. In line with the objective of this review and as patients with cardiovascular disease and cancer receive most of their treatment and interventions in hospitals, we aimed to include a hospital setting in this review. Studies were reviewed in accordance with the Preferred Reporting Items for Systematic Reviews and Meta-Analyses (PRISMA) guidelines [31]. PRISMA checklist can be found in the supplementary files. After verifying that no similar reviews exist, this review was registered in the international prospective register of systematic reviews, with PROSPERO ID: CRD42021229763 on February 6th, 2021.

### Data sources and search strategy

With the assistance of a specialist librarian, we developed search terms and conducted a systematic literature review in PubMed, Embase, CINAHL, and MEDLINE (OvidSP) databases. On January 9th, 2021, we conducted our initial search using subject headings related to “value-based healthcare”; “hospital”; “quality measures”; “outcome measures”; “patient-reported outcome measures”; “cardiovascular”; and “cancer”. Since papers related to certain key measures in VBHC were not found (e.g., “process measures”; “patient-reported experience measures” and “patient satisfaction”), we performed an additional search on February 15th, 2021. The initial selection was made based on the title and abstract, and the final selection was made based on the full-text reading. The reference lists of the included studies were scanned to search for additional potentially relevant articles. The complete search strategy is provided in the supplementary files.

### Eligibility criteria

We included studies that focused on process and outcome quality measures for cardiovascular diseases and cancer in the hospital VBHC context. Quantitative and qualitative studies that were published in English as full-text in the last 10 years were eligible for this review. We excluded studies that were published in a non-English language, unpublished/unindexed studies, review articles, gray literature, and studies published in “abstract” format. Additionally, studies that were conducted in a nonhospital setting, for diseases other than cardiovascular or cancer, or examined VBHC cost-related measures, were excluded as well.

### Selection process and data extraction

Initially, titles and abstracts were retrieved and uploaded into the bibliographic reference management software EndNote X9, and duplicates were removed. Two reviewers (RA, MH) independently screened the titles and abstracts and conducted a full-text review to assess the eligibility of the potentially relevant articles. Disagreements were resolved by discussion in the first place or consultation of a third reviewer (MP and WG) when discrepancies persisted. Excluded studies were listed along with their exclusion reasons in the supplementary files.

### Quality assessment

The methodological quality of the included studies was independently assessed by two reviewers (RA, MH) using four validated tools. The Critical Appraisal Skills Program (CASP) qualitative studies checklist (10 items), the National Institutes of Health (NIH) quality assessment tool for observational cohort and cross-sectional studies (14 items), and case-control studies (12 items) were used to assess the quality of qualitative and quantitative studies, respectively [32–34]. Guidance on Conducting and Reporting Delphi Studies (CREDES) was used to assess the quality of consensus studies that used a Delphi process (12 items) [35]. An additional 13th criterion was adopted by the reviewers for the fourth tool in order to fulfill the need to provide a description of ethical issues for the expert sample and research community [36]. All of these tools did not suggest a scoring system and did not provide the cut-offs for the overall quality rating. In this review, the total score of the methodological quality of each study was calculated based on the number of met items in the used tool. Then, the total scores were converted to percentages. Our research team agreed to unify the classification of studies and to divide the overall quality rating percentage into almost thirds. So, the studies were categorized as; “Good” for a score of 70–100%, “Fair” for 40–69%, and “Poor” for 0–39% [37].

### Data analysis

To better conceptualize quality measures related to VBHC, the Donabedian model that examines health services from three aspects: structure, process, and outcome, was adopted as a conceptual framework for this review combined with the three-tier Outcome Measures Hierarchy of Michael Porter [5, 6]. Deductive thematic analysis (analysis directed by a predefined concept model) was used with seven themes of quality measures covering process and outcome measures along with the risk adjustment variables [38]. Data extracted from each of the included studies were classified under one or multiple quality measures’ themes that included survival, degree of health or recovery, time to recovery and time to return to normal activities, disutility of care and treatment process, sustainability of health or recovery, long term consequences of therapy, and process measures. Using a data extraction form designed for this review, we extracted the following data from each included study: the objective of the study, research design, key findings, recommendations, limitations, used quality measures, and case-mix variables. The results are presented narratively and are supported with tables.

## Results

**Fig. 1:**
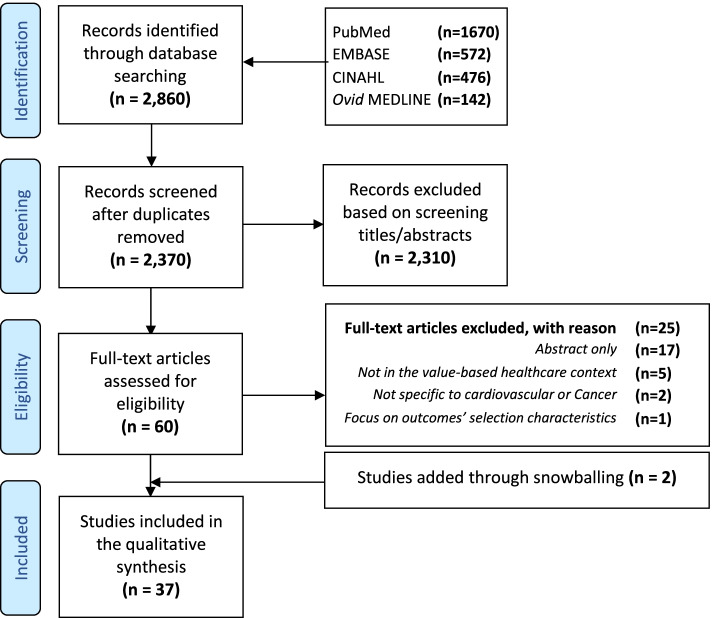
PRISMA flow diagram illustrating the study selection process. PRISMA, Preferred Reporting Items for Systematic Reviews and Meta-Analyses

The search strategy yielded 2860 publications. After screening for titles and abstracts, 60 articles were retrieved for full-text review. A total of 37 studies met our inclusion criteria; 2 of them were identified by screening the references of the included studies. A graphic representation of the search results is provided in Fig. 1.

### Features of the included studies

The general features of the included studies were summarized [39–75]. Detailed information is provided in the supplementary files. In line with the recent worldwide movement toward VBHC and outcome standardization efforts, 89% of the studies (*n* = 33) were published in 2015 or later. The number of publications that discussed quality measures in cardiovascular disease was 19. These studies addressed diagnoses such as coronary artery disease, heart failure, stroke and others such as hypertension, valve replacement and heart surgery. On the other hand, 18 studies addressed different types of cancer, especially breast cancer (*n* = 7), lung cancer (*n* = 4), prostate cancer (*n* = 3), colorectal, ovarian and pituitary cancers. Almost half of the studies (*n* = 17) were conducted in the USA, 7 studies in the Netherlands, and 4 studies in Germany, Taiwan, Belgium, and Spain. Some multi-country studies (*n* = 9) were conducted by the ICHOM. Cohort studies were employed in the majority of the papers (*n* = 17), while a consensus group was used in 13 studies. Other designs included mixed, cross-sectional, and qualitative methods. Among the studies that developed, used, or studied outcome measure sets, 20 studies validated these outcomes in clinical practice [39–41, 43, 44, 46–54, 60, 63, 65–67, 69].

### The overall quality of the studies

The results indicated that 18 studies were rated as good while 18 studies were rated as fair quality. Only one study was rated as poor quality. Limitations included the lack of clear rationale for using the Delphi technique, lack of pilot testing of the instruments, lack of description of ethical issues, lack of sample size justification, or blinding of outcome assessors.

### Themes

Quality measures extracted from the included studies were classified into seven themes; six themes for outcome measures and one theme for process measures. Detailed information is provided in the supplementary files. Despite including “quality measures” in the subject heading of the search, measures of the “structure” component of the Donabedian model were not found, restricting the findings to process and outcome measures only. Notably, the highest number of quality measures was found in the “disutility of care or treatment process” outcome theme (e.g., complications, side effects, treatment-related discomfort, errors and their consequences), while the lowest number of quality measures was found in the “long term consequences of therapy” outcome theme. Although our themes are collectively exhaustive, they are not mutually exclusive as the majority of studies (*n* = 32) examined quality measures at least in two separate themes rather than a single theme. The results for each of the themes are presented separately in the following sections.

### Survival

Survival measures (e.g., overall and disease-free survival) were commonly reported as measures of value by patients, clinicians, and other stakeholders involved in the development of the outcome sets in the included studies (*n* = 21) [39, 40, 42, 44, 49, 51, 55–60, 63–65, 68–71, 74, 75]. In addition, some studies reported that measuring all-cause mortality is superior to disease or cause-specific mortality, which is less meaningful to patients and harder to capture due to the non-availability or limited reliability of data related to the cause of death [42, 56, 57]. Administrative data (e.g., death registry or claims data) and clinician-reported data sources were used to collect survival/mortality-related information. Survival has become of increasing value and importance to patients over time and, accordingly, it was measured at different time points starting from 30 days post-index event (i.e., first clinical encounter) and extended up to 5 years, 10 years, or annually for life.

### Degree of health or recovery

As Quality of Life (QoL) is evaluated and reported by patients and includes measures that are relatively visible, their inclusion in the outcome sets of VBHC was very common, particularly in this theme (*n* = 24) [40, 41, 43, 44, 46, 52, 53, 56–59, 61–66, 70–76]. To ensure the inclusion of outcomes that the patients value most, the studies used a combination of generic and disease/condition-specific tools to measure QoL that covered multiple domains such as mental, functional, social, physical, and other aspects of health status. The Patient-Reported Outcomes Measurement Information System (PROMIS Global −10), Short Form-12, and Short Form-36 were used as generic tools in studies of cardiovascular disease [39, 40, 42, 43, 51, 53]. Additionally, the European Organization for Research and Treatment of Cancer–Core 30 (EORTC–C30) was used as a generic tool in studies of cancer disease [58, 60, 63, 69–72, 75]. In some studies (*n* = 3), the Patient Health Questionnaire was used as a complementary tool for the generic tools to measure anxiety and depression aspects [43, 56, 57]. The European Quality of Life (EuroQoL) tool was mainly used in studies of cancer [39, 60, 65, 72, 74, 75]. Longitudinal data and measurements of PROMs were used in almost all studies. In contrast to measuring QoL, 4 studies of cancer disease recommended measuring the quality of death by clinicians for patients at the end of life [58, 63, 71, 74]. PREMs were reported by one study in cardiovascular disease [49] and another study in cancer [61].

### Time to recover and time to return to normal activities

Patients often have particular concerns about the expected treatment and recovery times. This theme consists of outcomes that patients (across medical conditions) would value in terms of timeliness of recovery and fast return to normal activities. Five studies reported quality measures in this theme [39, 65, 68, 71, 74]. PROMs were used for outcomes like access to care and medications [39, 68] and time to recover and return to work [65]. At the same time, clinician-reported data were collected for treatment delay [68] and time from diagnosis to treatment [71, 74]. Longitudinal data collection was used for PROMs in this theme as well.

### Disutility of care or treatment process

In VBHC, it is important to measure the undesired outcomes of the treatment process that can affect the effectiveness and efficiency of care provided and consequently the value of the healthcare system. In line with this, most quality measures were captured in this theme. It included a wide spectrum of adverse events, side effects, and complications of the treatment and care process of cardiovascular and cancer patients. Clinician-reported information was the primary data source in all studies. Apparently, 4 studies reported surveillance bias with the current Venous Thromboembolism (VTE) outcome measure and recommended modifications for the public reporting and VBHC programs [46, 47, 53, 54].

### Sustainability of health or recovery

This theme includes what generally matters to patients in terms of maintaining achieved health outcomes and functional status that has a direct impact on value as a health outcome. Reported quality measures included readmissions, reoperations, disease recurrences, and other longer-term complications that were primarily reported by clinicians [39, 40, 42, 49, 51, 55, 60, 65, 68, 70]. Longitudinal data collection was used for these measures.

### Long-term consequences of therapy

Two studies, in pituitary and breast cancer, reported quality measures related to long-term consequences. In the first study, clinician-reported new pituitary deficiencies and accompanying replacement therapies [65]. In the second study, QoL measures were classified under this theme, not under the “degree of health or recovery”, because they were related to long-term consequences of therapy as reported in the study [68].

### Process measures

Despite the limited effect of process measures in value measurement, they were reported in 3 and 5 studies of cardiovascular and cancer diseases, respectively [46, 49, 55, 59, 61, 62, 66, 67]. In one study, a clinician-reported composite VTE prophylaxis measure was recommended over the current process and outcome VTE rate [46]. In another study, 12 process measures were recommended in addition to the currently measured outcome set for patients undergoing valve replacement procedures [55]. Other process measures monitored compliance with guidelines, treatment protocols, care coordination, timely assessment, and documentation. Clinician-reported measures were adopted for the majority of process measures. These studies considered process measures as enablers to improve outcomes and to establish the foundation for improving value in VBHC models.

### Case-mix variables

The reported case-mix variables can be clustered into three main groups: demographics (e.g., age, sex, race/ethnicity, educational level), baseline clinical factors, and comorbidities (e.g., body mass index, family history, medical conditions, diabetes mellitus, hypertension, renal failure) as well as treatment variables (e.g., medications, surgeries, interventions). Measuring these variables at baseline (i.e., first clinical encounter) was used in all studies that measured them [39, 41–44, 46–51, 54, 57–66, 68, 70–72, 74, 75]. Another two studies recommended including frailty score and stroke severity in risk adjustment for heart failure and acute ischemic stroke patients, respectively [41, 48].

### Data sources

In our review, it was found that administrative data were the least frequently used source for outcome measurement, and they were limited to measures such as survival and mortality [40, 41, 43, 45, 50, 52, 56–61, 64–66, 69–72, 75, 76]. Data reported by clinicians and patients were more frequently used in the studies reviewed.

## Discussion

In VBHC, standard outcome sets are developed to cover the entire care cycle rather than individual services or interventions for a medical condition [5, 8]. Quality and outcome measurement starts at the first encounter in primary care or in hospital, and may extend to out-hospital and long-term follow-up of outcomes and QoL [40, 51, 55, 60, 63, 65, 69, 75]. To enhance comparisons of these outcome sets across different settings, the studies in this review highlighted two factors: accurate risk adjustment procedures and standardization of definitions and measurement of a sufficient minimum set of measures [39, 42, 48, 51, 55–58, 60, 70, 71].

As indicated by our review, despite the heterogeneity in outcome measures for different medical conditions in cardiovascular diseases and cancer, there is consensus to include measures such as survival rates and disease control, disutility of care, and QoL. The recommended outcome sets must be validated in clinical practice and must go through regular maintenance cycles to ensure that the most relevant set of measures is included with the necessary improvement and refinement procedures based on the results of the measures, feedback from users and stakeholders, and the recommended scientific evidence [40, 51, 75].

Our review identified that evidence is mixed on processes versus health outcomes in VBHC models. Some studies reported that process measures are good predictors of health outcomes [55] and improving processes is necessary to improve outcomes with relatively easier data collection, benchmarking, and risk adjustment that can be fostered by the availability of continuous and immediate clinician-reported information [62]. On the other hand, publications reported that measuring health outcomes is what matters most to the patients by focusing on clinical outcomes and PROMs regardless of what processes were followed to lead to those outcomes (considering that one outcome can be the result of multiple care processes at a time) [56, 58, 60, 62–64, 68, 75]. As process measures are not substitutes for outcome measures [5], other approaches support a combination of both measures. Indeed, although process measures are still used in different public reporting programs around the world [44], evidence is mounting on measuring patient-relevant outcomes that are reported by the patient, proxy, or clinician [1, 5]..

Data for either process or outcome measures are obtained via multiple data sources. Previous studies indicated that using easy-to-find administrative (e.g., death registry and claims data) and billing data for quality and performance improvement is insufficient and must be complemented by clinician- and patient-reported measures [5]. Despite considering clinician- and patient-reported outcomes as robust data sources, developing a rigorous and longitudinal data collection and measurement infrastructure is a challenge in different organizations due to the associated administrative burden of data collection and management [5]. For instance, in the USA, it was found that an average of 785 hours per physician and more than $15.4 billion were spent on reporting quality measures [76]. Therefore, it is important that the measures adequately evaluate quality, cost, and patient outcomes but they should not increase the burden of data collection and reporting on healthcare organizations. Hence, integrated quality management systems that utilize health information technologies and web-based applications are recommended to facilitate the operational aspects of data collection and automate the questionnaire-administration process for different PROMs instruments [42, 57, 75, 77–79].

Interestingly, patients were involved in the development of the standardized outcome sets to ensure that their perspectives were incorporated into measures development [39, 42, 45, 55–58, 60, 64, 68–71, 74, 75]. Relevant PROMs can orient the patients about the potential outcomes of their medical condition and, at the same time, solicit feedback on their needs and priorities regarding the delivered treatment modalities. Thus, PROMs can facilitate mutual communication between patients and their healthcare providers and consequently support the shared decision-making process and patient-centered care [40, 52, 58, 60, 63–65, 69, 70, 72, 73, 75, 80]. Our results were consistent with those of previous studies that recommended using both generic and disease-specific instruments simultaneously to measure PROMs [43, 77, 81, 82]. The generic tools facilitate comparisons across conditions, while disease-specific tools have greater face validity and credibility and guide improvements at the disease level [83].

Accordingly, measuring PROMs, in addition to systematic and multidimensional clinical outcomes, prioritizes value and enables benchmarking, improvement, learning, and sharing of best practices across different settings [42, 44, 51, 63, 64, 75]. These findings were emphasized in our review through a balanced approach of obtaining patient perspectives of care (i.e., PROMs and PREMs) along with clinical outcomes for VBHC. Notably, PREMs were discussed in only two studies. Further research is recommended to gain more insights into the recommended practice of integrating PREMs in VBHC initiatives.

This study should be reviewed considering some limitations. First, a consensus group was used in 14 (39%) of the included studies in this review. This research design has a lower grade of evidence than randomized clinical trials that provide high-quality evidence to guide clinical practice [4, 42, 64, 75]. Second, the reported outcome sets must be validated in clinical practice and among patients, as being relevant, to enhance their feasibility and acceptance across diverse healthcare systems around the world. Third, although our review was carefully designed, we cannot completely exclude publication bias, given that relevant studies might not have been published at the time of the review, as well as selection bias since we only reviewed English-language literature.

This review adds to the literature on VBHC by summarizing quality measures used in the top deadliest diseases in the world and highlighting the recommended quality measurement practices that inform the new VBHC reform in healthcare systems.

## Conclusion

The transformation to the VBHC is progressing at different levels in various countries and organizations around the world. Defining, measuring, reporting, and comparing outcomes are essential steps in integrating VBHC principles within healthcare systems. This systematic review identified quality measures, tools, and data sources used in VBHC for cardiovascular diseases and cancer in hospitals. It highlights the practice of including clinical outcomes and PROMs in the standard sets of measures to assess “value” from the patients’ perspectives that can differ from what clinicians value in healthcare. Hospitals that started the development of VBHC initiatives or are planning to do so can choose whether they prefer to implement the standardized outcome step-by-step, collect additional measures, or develop their own set. However, they need to ensure that their performance can be consistently compared to that of their peers and that they measure what prioritizes and maximizes value for their patients.

## Supplementary Information

**Additional file 1.**

**Additional file 2.**

**Additional file 3.**

**Additional file 4.**

**Additional file 5.**

## Data Availability

All data generated or analyzed during this study are included in this published article and its supplementary information files.

## Abbreviations

VBHC: Value-Based Healthcare
ICHOM: International Consortium for Healthcare Outcome Measurement
OECD: Organization of Economic Co-operation and Development
VBHCD: Value-Based Health Care Delivery
NHS: The National Health Service
PROMs: Patient-Reported Outcome Measures
PREMs: Patient-Reported Experience Measures
PRISMA: Preferred Reporting Items for Systematic Reviews and Meta-Analyses
CASP: Critical Appraisal Skills Program
NIH: National Institutes of Health
CREDES: Guidance on Conducting and Reporting Delphi Studies
QoL: Quality of Life
PROMIS Global −10: The Patient-Reported Outcomes Measurement Information System
EORTC–C30: The European Organization for Research and Treatment of Cancer–Core 30
EuroQoL: The European Quality of Life
VTE: Venous Thromboembolism
