# Supplementary material for: Quality measurement for cardiovascular diseases and cancer in hospital value-based healthcare: a systematic review of the literature
Source: BMC Health Serv Res. 2022 Aug 1;22:979. doi: 10.1186/s12913-022-08347-x (PMC9341062; doi:10.1186/s12913-022-08347-x)
Supplement: Supplementary file 2 — Additional file 2. [file 12913_2022_8347_MOESM2_ESM.pdf]

Additional File	
**Article title:**	Quality Measurement for Cardiovascular Diseases and Cancer in Hospital Value-Based Healthcare: A Systematic Review of the Literature.
**Author names:**	Rawia Abdalla, Milena Pavlova, Mohammed Hussein, and Wim Groot.
**Affiliation and e-mail address of the corresponding author:**	Maastricht University, Department of Health Services Research, CAPHRI, Maastricht University Medical Center, Faculty of Health, Medicine and Life Sciences, Maastricht, Limburg, The Netherlands.<br>r.abdalla@maastrichtuniversity.nl<br>Rawia85@yahoo.com
**Caption:**	This file includes all search strategies in the databases.

Additional File 2:

ALL SEARCH STRATEGIES

CINAHL, PubMed, EMBASE, and** Ovid **MEDLINE

Initial Search 09. January. 2021

PubMed NCBI

Searched via PubMed - NCBI (https://www.ncbi.nlm.nih.gov/pubmed/).
Searched on 09 January 2021
Records retrieved: **1,315**

(((("value-based"[All Fields] OR "pay for performance"[All Fields] OR "P4P"[All Fields] OR "reimbursement, incentive"[MeSH Terms] OR "reimbursement, incentive"[All Fields] OR "reimbursement"[All Fields] OR "payment"[All Fields] OR "value driven"[All Fields] OR "financial* reward*"[All Fields] OR "financial* incentive*"[All Fields]) **AND** ("hospitals"[MeSH Terms] OR "hospitals"[All Fields] OR "hospital"[All Fields] OR "center"[All Fields] OR "centre"[All Fields] OR "health facility"[All Fields] OR "health organization"[All Fields])) **AND** ("patient reported outcome measures"[MeSH Terms] OR "patient reported outcome measures"[All Fields] OR "patient reported outcome"[All Fields] OR "patient centered"[All Fields] OR "quality measurement"[All Fields] OR "performance measurement"[All Fields] OR "measurement of quality"[All Fields] OR "quality measures"[All Fields] OR "performance measures"[All Fields] OR "quality indicators"[All Fields] OR "performance indicators"[All Fields] OR "quality metrics"[All Fields] OR "performance metrics"[All Fields] OR "key performance indicators"[All Fields] OR "process measure*"[All Fields] OR "process indicator"[All Fields] OR "process assessment, health care"[MeSH Terms] OR "process assessment, health care"[All Fields] OR "value measures"[All Fields] OR "value indicators"[All Fields] OR "value metrics"[All Fields] OR "quality rate"[All Fields] OR "performance rate"[All Fields] OR "quality rating"[All Fields] OR "performance rating"[All Fields] OR "total performance score"[All Fields] OR "clinical measure"[All Fields] OR "outcome measure"[All Fields] OR "clinical indicator"[All Fields] OR "outcome indicator"[All Fields] OR "clinical metric"[All Fields] OR "outcome metric"[All Fields] OR "patient reported measure"[All Fields] OR "clinical outcome"[All Fields] OR "outcome assessment, health care"[MeSH Terms] OR "outcome assessment, health care"[All Fields] OR "patient outcome assessment"[MeSH Terms] OR "patient outcome assessment"[All Fields])) AND ("cardiovascular diseases"[MeSH Terms] OR "cardiovascular diseases"[All Fields] OR "cardio*"[All Fields] OR "heart"[All Fields] OR "neoplasms"[MeSH Terms] OR "cancer"[All Fields] OR "oncology"[All Fields] OR "oncology service, hospital"[MeSH Terms] OR "oncology service, hospital"[All Fields])) **AND** "loattrfull text"[sb] **AND** eng[la] **AND** ("2010/01/01"[PDAT] : "2021/01/09"[PDAT])

Search Details

Number	Query	Results
#72	#71 AND "2010/01/01"[PDAT] : "2021/01/09"[PDAT]	**1,315**
#71	#70 AND eng[la]	1,590
#70	#69 AND "loattrfull text"[sb]	1,609
#69	#11 AND #19 AND #58 AND #68	1,686
#68	#59 OR #60 OR #61 OR #62 OR #63 OR #64 OR #65 OR #66 OR #67	8,299,664
#67	"oncology service, hospital"[All Fields]	1,480
#66	"oncology service, hospital"[MeSH Terms]	1,466
#65	"oncology"[All Fields]	673,379
#64	"cancer"[All Fields]	2,391,210
#63	"neoplasms"[MeSH Terms]	3,389,178
#62	"heart"[All Fields]	1,459,503
#61	"cardio*"[All Fields]	2,656,355
#60	"cardiovascular diseases"[All Fields]	211,793

#59	"cardiovascular diseases"[MeSH Terms]	2,416,462
#58	#20 OR #21 OR #22 OR #23 OR #24 OR #25 OR #26 OR #27 OR #28 OR #29 OR #30 OR #31 OR #32 OR #33 OR #34 OR #35 OR #36 OR #37 OR #38 OR #39 OR #40 OR #41 OR #42 OR #43 OR #44 OR #45 OR #46 OR #47 OR #48 OR #49 OR #50 OR #51 OR #52 OR #53 OR #54 OR #55 OR #56 OR #57	1,354,643
#57	"patient outcome assessment"[All Fields]	5,416
#56	"patient outcome assessment"[MeSH Terms]	12,427
#55	"outcome assessment, health care"[All Fields]	74,681
#54	"outcome assessment, health care"[MeSH Terms]	1,157,585
#53	"clinical outcome"[All Fields]	77,952
#52	"patient reported measure"[All Fields]	159
#51	"outcome metric"[All Fields]	124
#50	"clinical metric"[All Fields]	43
#49	"outcome indicator"[All Fields]	362
#48	"clinical indicator"[All Fields]	917
#47	"outcome measure"[All Fields]	63,900
#46	"clinical measure"[All Fields]	801
#45	"total performance score"[All Fields]	51
#44	"performance rating"[All Fields]	247
#43	"quality rating"[All Fields]	730
#42	"performance rate"[All Fields]	207
#41	"quality rate"[All Fields]	47
#40	"value metrics"[All Fields]	41
#39	"value indicators"[All Fields]	19
#38	"value measures"[All Fields]	74
#37	"process assessment, health care"[All Fields]	32,389
#36	"process assessment, health care"[MeSH Terms]	4,784
#35	"process indicator"[All Fields]	115
#34	"process measure*"[All Fields]	2,507
#33	"key performance indicators"[All Fields]	721
#32	"performance metrics"[All Fields]	2,529
#31	"quality metrics"[All Fields]	2,176
#30	"performance indicators"[All Fields]	3,427
#29	"quality indicators"[All Fields]	20,487
#28	"performance measures"[All Fields]	7,404
#27	"quality measures"[All Fields]	4,569
#26	"measurement of quality"[All Fields]	686
#25	"performance measurement"[All Fields]	1,676
#24	"quality measurement"[All Fields]	1,950
#23	"patient centered"[All Fields]	33,256
#22	"patient reported outcome"[All Fields]	14,401
#21	"patient reported outcome measures"[All Fields]	10,542
#20	"patient reported outcome measures"[MeSH Terms]	6,974
#19	#12 OR #13 OR #14 OR #15 OR #16 OR #17 OR #18	9,131,902
#18	"centre"[All Fields]	1,473,063
#17	"center"[All Fields]	3,907,180
#16	"health organization"[All Fields]	103,662

#15	"health facility"[All Fields]	27,447
#14	"hospital"[All Fields]	5,023,693
#13	"hospitals"[All Fields]	579,989
#12	"hospitals"[MeSH Terms]	278,634
#11	#1 OR #2 OR #3 OR #4 OR #5 OR #6 OR #7 OR #8 OR #9 OR #10	76,876
#10	"financial* incentive*"[All Fields]	4,721
#9	"financial* reward*"[All Fields]	516
#8	"value driven"[All Fields]	744
#7	"payment"[All Fields]	28,563
#6	"reimbursement"[All Fields]	45,300
#5	"reimbursement, incentive"[All Fields]	4,456
#4	"reimbursement, incentive"[MeSH Terms]	4,440
#3	"P4P"[All Fields]	523
#2	"pay for performance"[All Fields]	2,118
#1	"value-based"[All Fields]	6,819

EMBASE and Ovid **MEDLINE**(R)

Searched via **Ovid** (http://ovidsp.ovid.com/).
Date range searched: 2010 to current.
Searched on 09 January 2021
EMBASE Records retrieved: **249**
MEDLINE (R) Records retrieved: **95**

Number	Searches	EMBASE Results	MEDLINE Results
1	value-based.mp.	8176	5007
2	exp reimbursement/	58164	0
3	reimbursement.mp.	70061	40598
4	(pay for performance or P4P).mp.	2930	2068
5	(Reimbursement adj2 incentive).mp.	92	4487
6	payment$1.tw.	36528	25691
7	value driven.mp.	435	271
8	financial$ reward$.mp.	754	527
9	financial$ incentive$.mp.	5822	4089
10	or/1-9	110915	67218
11	exp hospital/	1160533	278358
12	hospital$1.mp.	2366566	1291872
13	healthcare organi#ation$.tw.	7361	5560
14	health$ institution$.mp.	7624	4676
15	exp health care facility/	1574703	0
16	health$ facilit$.tw.	25915	16096
17	exp cancer center/	38027	0
18	((cancer or oncology) adj2 (center$1 or centre$1 or institut$ or facilit$)).tw.	86413	38716

19	((cardiac or heart or cardio$) adj2 (center$1 or centre$1 or institut$ or facilit$)).tw.	19856	8739
20	or/11-19	3027099	1360001
21	exp patient-reported outcome/	26784	6918
22	patient reported outcome.mp.	33489	11207
23	patient reported outcome measures.mp.	6977	8829
24	patient centered.mp.	25108	28315
25	(quality rating$1 or quality indicator$1 or quality measur$ or quality metric$1 or quality rate$1).mp.	31278	28175
26	(performance measure$ or performance indicator$1 or performance metric$1 or performance rat$3).mp.	29775	15473
27	key performance indicators.mp.	1339	519
28	process assessment.mp.	1117	32543
29	process measure$1.mp.	3177	1855
30	process indicators.mp.	1105	726
31	(value measur$ or value indicator$1 or value metric$1).mp.	2287	1344
32	(clinical metric$1 or clinical measur$ or clinical indicator$1).mp.	22722	12053
33	exp outcome assessment/	576114	0
34	(outcome measur$ or outcome indicator$1 or outcome metric$1 or outcome assessment or clinical outcome$1).mp.	1099168	415605
35	(process measur$ or process indicator$1 or process metric$1).mp.	4984	2930
36	process assessment.mp.	1117	32543
37	process assessment, health care.mp.	430	32301
38	patient reported measure$1.mp.	1140	542
39	total performance score.mp.	59	44
40	patient outcome assessment.mp.	988	5230
41	or/21-40	1218967	516956
42	exp cardiovascular disease/	4161544	2414394
43	cardiovascular diseases.mp.	84286	179111
44	cardio$.mp.	1725975	887962
45	heart/	323578	147787
46	exp neoplasm/	4583128	3386512
47	neoplasms.mp.	234703	2699661
48	exp malignant neoplasm/	3542517	3386512
49	malignant neoplasm.mp.	74419	4070
50	cancer.mp.	3685371	1515370
51	exp oncology/ or oncolog$.mp. or oncology.mp.	414108	146332
52	oncology service, hospital.mp.	24	1473
53	(oncology adj2 service$1).mp.	3504	2709
54	or/42-53	9505287	6224028
55	10 and 20 and 41 and 54	1776	536
56	limit 55 to yr="2010 -Current"	1578	379
57	limit 56 to english language	1566	370
58	limit 57 to full text	**249**	**95**

EBSCO CINAHL

Cumulative Index to Nursing and Allied Health Literature (CINAHL)

Searched via EBSCOhost (www.ebscohost.com/).
Searched on 09 January 2021
Records retrieved: **368**

Number	Query	Results
S42	S41 Narrow by Language: - english	**368**
S41	S40 Limiters - Published Date: 01.01.2010 - 31.01.2021	397
S40	S10 AND S18 AND S32 AND S39	534
S39	S33 OR S34 OR S35 OR S36 OR S37 OR S38	1,445,275
S38	TX "neoplasm*" OR "oncology" OR "cancer" OR	631,382
S37	MH "oncology+"	11,218
S36	MH "neoplasms+"	558,539
S35	TI "cardi*" OR AB "cardi*" OR TI "heart" OR AB "heart"	408,900
S34	TX "cardiovascular disease*"	99,941
S33	MH "cardiovascular diseases+"	598,405
S32	S19 OR S20 OR S21 OR S22 OR S23 OR S24 OR S25 OR S26 OR S27 OR S28 OR S29 OR S30 OR S31	223,113
S31	TX "patient reported measure" OR "patient outcome assessment"	16,103
S30	TX "outcome measur*" OR "outcome metric*" OR "outcome indicator*" OR "outcome assessment"	133,728
S29	MH "patient-reported outcomes+"	2,837
S28	MH "outcome assessment"	44,531
S27	TX "clinical measur*" OR "clinical metric*" OR "clinical indicator*" OR "clinical outcome*"	71,599
S26	MH "clinical indicators"	12,543
S25	TX "value measur*" OR "value metric*" OR "value indicator*"	272
S24	TX "process measur*" OR " process assessment" OR "process indicator*"	6,415
S23	MH "process assessment (health care)+"	8,173
S22	TX "performance measur*" OR "performance metric*" OR "performance rat*" OR "performance indicator*" OR "total performance score"	11,340
S21	TX "key performance indicators"	410
S20	MH "performance measurement systems"	2,263
S19	TX "quality measur*" OR "quality metric*" OR "quality rat*" OR "quality indicator*"	14,679
S18	S11 OR S12 OR S13 OR S14 OR S15 OR S16 OR S17	2,436,037
S17	TI ((cancer or oncology) N2 (center OR centers OR centre OR centres OR institute* OR facilit* OR "hospital unit*")) OR AB ((cancer or oncology) N2 (center OR centers OR centre OR centres OR institute* OR facilit* OR "hospital unit*"))	16,765
S16	(MH "Cancer Care Facilities")	5,642
S15	TI ((cardiac or heart or cardio*) N2 (center OR centers OR centre OR centres OR institute* OR facilit* OR "hospital unit*")) OR AB ((cardiac or heart or cardio*) N2 (center OR centers OR centre OR centres OR institute* OR facilit* OR "hospital unit*")) OR TI "coronary care unit*" OR AB "coronary care unit*"	4,106
S14	(MH "Academic Medical Centers")	74,879
S13	TX "center" OR "centers" OR "centre" OR "centres"	1,271,749
S12	TX "hospital" OR "hospitals"	1,594,487
S11	MH "Hospitals+"	118,672
S10	S1 OR S2 OR S3 OR S4 OR S5 OR S6 OR S7 OR S8 OR S9	63,594
S9	TX "financial* incentive*"	2,876

S8	TX "financial* reward*"	368
S7	TI "payment" OR AB "payment"	13,641
S6	TX reimbursement	47,458
S5	MH "Reimbursement, Incentive"	2,634
S4	TX "pay for performance" OR P4P	1,898
S3	TX "value-driven"	485
S2	(MH "Value-Based Health Care") OR (MH "Value-Based Insurance") OR (MH "Value-Based Purchasing")	1,580
S1	TX value-based	5,671

Second Search 15. February. 2021

PubMed NCBI

Searched via PubMed - NCBI (https://www.ncbi.nlm.nih.gov/pubmed/).
Searched on 15 February 2021... Records retrieved: **1,670**

(((("value-based"[All Fields] OR "pay for performance"[All Fields] OR "P4P"[All Fields] OR "reimbursement, incentive"[MeSH Terms] OR "reimbursement, incentive"[All Fields] OR "reimbursement"[All Fields] OR "payment"[All Fields] OR "value driven"[All Fields] OR "financial* reward*"[All Fields] OR "financial* incentive*"[All Fields] OR "international consortium"[All Fields]) AND ("hospitals"[MeSH Terms] OR "hospitals"[All Fields] OR "hospital"[All Fields] OR "center"[All Fields] OR "centre"[All Fields] OR "health facility"[All Fields] OR "health organization"[All Fields])) **AND** ("patient reported outcome measures"[MeSH Terms] OR "patient reported outcome measures"[All Fields] OR "patient reported outcome"[All Fields] OR "quality measurement"[All Fields] OR "performance measurement"[All Fields] OR "measurement of quality"[All Fields] OR "quality measures"[All Fields] OR "performance measures"[All Fields] OR "quality indicators"[All Fields] OR "performance indicators"[All Fields] OR "quality metrics"[All Fields] OR "performance metrics"[All Fields] OR "key performance indicators"[All Fields] OR "process measure*"[All Fields] OR "process indicator"[All Fields] OR "process assessment, health care"[MeSH Terms] OR "process assessment, health care"[All Fields] OR "value measures"[All Fields] OR "value indicators"[All Fields] OR "value metrics"[All Fields] OR "quality rate"[All Fields] OR "performance rate"[All Fields] OR "quality rating"[All Fields] OR "performance rating"[All Fields] OR "total performance score"[All Fields] OR "clinical measure"[All Fields] OR "outcome measure"[All Fields] OR "clinical indicator"[All Fields] OR "outcome indicator"[All Fields] OR "clinical metric"[All Fields] OR "outcome metric"[All Fields] OR "patient reported measure"[All Fields] OR "clinical outcome"[All Fields] OR "outcome assessment, health care"[MeSH Terms] OR "outcome assessment, health care"[All Fields] OR "patient outcome assessment"[MeSH Terms] OR "patient outcome assessment"[All Fields] OR "patient satisfaction"[MeSH Terms] OR "patient satisfaction"[All Fields] OR "patient preference"[MeSH Terms] OR "patient preference"[All Fields] OR "patient-centered care"[MeSH Terms] OR "patient-centered care"[All Fields] OR "patient participation"[MeSH Terms] OR "patient participation"[All Fields] OR "patient experience"[All Fields] OR "patient reported experience measures"[All Fields] OR "PREMs"[All Fields] OR "patient centeredness"[All Fields] OR "patient centered"[All Fields])) **AND** ("cardiovascular diseases"[MeSH Terms] OR "cardiovascular diseases"[All Fields] OR "cardio*"[All Fields] OR "heart"[All Fields] OR "neoplasms"[MeSH Terms] OR "cancer"[All Fields] OR "oncology"[All Fields] OR "oncology service, hospital"[MeSH Terms] OR "oncology service, hospital"[All Fields])) **AND** "loattrfull text"[sb] **AND** eng[la] **AND** ("2010/01/01"[PDAT] : "2021/02/15"[PDAT])

Search Details

Number	Query	Results
#85	#84 AND "2010/01/01"[PDAT] : "2021/02/15"[PDAT]	1,670
#84	#83 AND eng[la]	1,987
#83	#82 AND "loattrfull text"[sb]	2,002
#82	#12 AND #20 AND #71 AND #81	2,107
#81	#72 OR #73 OR #74 OR #75 OR #76 OR #77 OR #78 OR #79 OR #80	8,388,885
#80	"oncology service, hospital"[All Fields]	1,500
#79	"oncology service, hospital"[MeSH Terms]	1,485
#78	"oncology"[All Fields]	664,815
#77	"cancer"[All Fields]	2,430,924
#76	"neoplasms"[MeSH Terms]	3,420,442
#75	"heart"[All Fields]	1,475,175
#74	"cardio*"[All Fields]	2,684,878
#73	"cardiovascular diseases"[All Fields]	215,435
#72	"cardiovascular diseases"[MeSH Terms]	2,455,154
#71	#21 OR #22 OR #23 OR #24 OR #25 OR #26 OR #27 OR #28 OR #29 OR #30 OR #31 OR #32 OR #33 OR #34 OR #35 OR #36 OR #37 OR #38 OR	1,476,344

	#39 OR #40 OR #41 OR #42 OR #43 OR #44 OR #45 OR #46 OR #47 OR #48 OR #49 OR #50 OR #51 OR #52 OR #53 OR #54 OR #55 OR #56 OR #57 OR #58 OR #59 OR #60 OR #61 OR #62 OR #63 OR #64 OR #65 OR #66 OR #67 OR #68 OR #69 OR #70	
#70	"patient centered"[All Fields]	34,529
#69	"patient centeredness"[All Fields]	1,122
#68	"PREMs"[All Fields]	146
#67	"patient reported experience measures"[All Fields]	126
#66	"patient experience"[All Fields]	7,057
#65	"patient participation"[All Fields]	28,460
#64	"patient participation"[MeSH Terms]	26,727
#63	"patient-centered care"[All Fields]	23,597
#62	"patient-centered care"[MeSH Terms]	21,162
#61	"patient preference"[All Fields]	13,090
#60	"patient preference"[MeSH Terms]	9,126
#59	"patient satisfaction"[All Fields]	103,244
#58	"patient satisfaction"[MeSH Terms]	92,162
#57	"patient outcome assessment"[All Fields]	5,525
#56	"patient outcome assessment"[MeSH Terms]	13,211
#55	"outcome assessment, health care"[All Fields]	75,630
#54	"outcome assessment, health care"[MeSH Terms]	1,173,459
#53	"clinical outcome"[All Fields]	79,149
#52	"patient reported measure"[All Fields]	165
#51	"outcome metric"[All Fields]	124
#50	"clinical metric"[All Fields]	44
#49	"outcome indicator"[All Fields]	369
#48	"clinical indicator"[All Fields]	936
#47	"outcome measure"[All Fields]	64,795
#46	"clinical measure"[All Fields]	810
#45	"total performance score"[All Fields]	52
#44	"performance rating"[All Fields]	248
#43	"quality rating"[All Fields]	739
#42	"performance rate"[All Fields]	208
#41	"quality rate"[All Fields]	49
#40	"value metrics"[All Fields]	42
#39	"value indicators"[All Fields]	20
#38	"value measures"[All Fields]	74
#37	"process assessment, health care"[All Fields]	32,692
#36	"process assessment, health care"[MeSH Terms]	4,829
#35	"process indicator"[All Fields]	115
#34	"process measure*"[All Fields]	2,545
#33	"key performance indicators"[All Fields]	755
#32	"performance metrics"[All Fields]	2,615
#31	"quality metrics"[All Fields]	2,251
#30	"performance indicators"[All Fields]	3,523
#29	"quality indicators"[All Fields]	20,753

#28	"performance measures"[All Fields]	7,669
#27	"quality measures"[All Fields]	4,673
#26	"measurement of quality"[All Fields]	696
#25	"performance measurement"[All Fields]	1,691
#24	"quality measurement"[All Fields]	2,022
#23	"patient reported outcome"[All Fields]	15,312
#22	"patient reported outcome measures"[All Fields]	11,333
#21	"patient reported outcome measures"[MeSH Terms]	7,587
#20	#13 OR #14 OR #15 OR #16 OR #17 OR #18 OR #19	9,309,688
#19	"centre"[All Fields]	1,509,191
#18	"center"[All Fields]	3,998,666
#17	"health organization"[All Fields]	106,333
#16	"health facility"[All Fields]	27,693
#15	"hospital"[All Fields]	5,125,107
#14	"hospitals"[All Fields]	589,586
#13	"hospitals"[MeSH Terms]	281,442
#12	#1 OR #2 OR #3 OR #4 OR #5 OR #6 OR #7 OR #8 OR #9 OR #10 OR #11	78,124
#11	"international consortium"[All Fields]	1,436
#10	"financial* incentive*"[All Fields]	4,887
#9	"financial* reward*"[All Fields]	541
#8	"value driven"[All Fields]	767
#7	"payment"[All Fields]	29,014
#6	"reimbursement"[All Fields]	45,802
#5	"reimbursement, incentive"[All Fields]	4,486
#4	"reimbursement, incentive"[MeSH Terms]	4,470
#3	"P4P"[All Fields]	531
#2	"pay for performance"[All Fields]	2,151
#1	"value-based"[All Fields]	7,016

EMBASE and Ovid **MEDLINE**(R)

Searched via **Ovid** (http://ovidsp.ovid.com/).
Date range searched: 2010 to current.
Searched on 15 February 2021
EMBASE Records retrieved: **572**
MEDLINE (R) Records retrieved: **142**

Number	Searches	EMBASE Results	MEDLINE results
1	value-based.mp.	8430	5171
2	exp reimbursement/	58918	0
3	reimbursement.mp.	71012	40934
4	(pay for performance or P4P).mp.	2971	2079
5	(Reimbursement adj2 incentive).mp.	92	4514
6	payment$1.tw.	37235	25998
7	value driven.mp.	442	281
8	financial$ reward$.mp.	762	536
9	financial$ incentive$.mp.	5937	4140
10	international consortium.mp.	1715	1006
11	or/1-10	112758	67982
12	exp hospital/	1181692	281175
13	hospital$1.mp.	2415735	1308634
14	healthcare organi#ation$.tw.	7524	5639
15	health$ institution$.mp.	7835	4795
16	exp health care facility/	1605002	0
17	health$ facilit$.tw.	26766	16622
18	exp cancer center/	38860	0
19	((cancer or oncology) adj2 (center$1 or centre$1 or institut$ or facilit$)).tw.	88126	39297
20	((cardiac or heart or cardio$) adj2 (center$1 or centre$1 or institut$ or facilit$)).tw.	20227	8889
21	or/12-20	3090103	1378016
22	exp patient-reported outcome/	28625	7522
23	patient reported outcome.mp.	35341	11937
24	patient reported outcome measures.mp.	7375	9492
25	(quality rating$1 or quality indicator$1 or quality measur$ or quality metric$1 or quality rate$1).mp.	31903	28605
26	(performance measure$ or performance indicator$1 or performance metric$1 or performance rat$3).mp.	30325	15777
27	key performance indicators.mp.	1400	538
28	process assessment.mp.	1127	32864
29	process measure$1.mp.	3235	1876
30	process indicators.mp.	1119	732
31	(value measur$ or value indicator$1 or value metric$1).mp.	2310	1354

32	(clinical metric$1 or clinical measur$ or clinical indicator$1).mp.	23142	12246
33	exp outcome assessment/	589519	0
34	(outcome measur$ or outcome indicator$1 or outcome metric$1 or outcome assessment or clinical outcome$1).mp.	1121524	422658
35	(process measur$ or process indicator$1 or process metric$1).mp.	5066	2960
36	process assessment.mp.	1127	32864
37	process assessment, health care.mp.	431	32615
38	patient reported measure$1.mp.	1168	562
39	total performance score.mp.	59	44
40	patient outcome assessment.mp.	1001	5333
41	exp patient satisfaction/	144462	92037
42	patient satisfaction.mp.	154249	96126
43	exp patient preference/	20067	9095
44	patient preference.mp.	23699	12048
45	"patient centered$".mp.	26726	29230
46	exp patient participation/	28746	26683
47	patient participation.mp.	30484	27783
48	patient experience.mp.	12221	4923
49	patient reported experience measures.mp.	197	80
50	PREMs.mp.	221	72
51	or/22-50	1414432	634810
52	exp cardiovascular disease/	4221616	2434305
53	cardiovascular diseases.mp.	86033	181751
54	cardio$.mp.	1750818	898431
55	heart/	324886	148399
56	exp neoplasm/	4645097	3416393
57	neoplasms.mp.	237524	2724802
58	exp malignant neoplasm/	3594121	3416393
59	malignant neoplasm.mp.	79077	4100
60	cancer.mp.	3745258	1537295
61	exp oncology/ or oncolog$.mp. or oncology.mp.	420723	149192
62	oncology service, hospital.mp.	24	1494
63	(oncology adj2 service$1).mp.	3626	2755
64	or/52-63	9635580	6280246
65	11 and 21 and 51 and 64	2078	599
66	limit 65 to yr="2010 -Current"	1835	420
67	limit 66 to English language	1821	409
68	limit 67 to full text	**572**	**142**

EBSCO CINAHL

Cumulative Index to Nursing and Allied Health Literature (CINAHL)

Searched via EBSCOhost (www.ebscohost.com/).
Searched on 15 February 2021.
Records retrieved: **476**

Number	Query	Results
S52	S51 Limiters - Source Types: Academic Journals	**476**
S51	S50 Limiters - Language: English	515
S50	S49 Limiters - Published Date: 01.01.2010 - 15.02.2021	517
S49	S11 AND S19 AND S41 AND S48	677
S48	S42 OR S43 OR S44 OR S45 OR S46 OR S47	1,465,881
S47	TX "neoplasm*" OR "oncology" OR "cancer" OR	641,845
S46	MH "oncology+"	11,374
S45	MH "neoplasms+"	567,356
S44	TI "cardi*" OR AB "cardi*" OR TI "heart" OR AB "heart"	415,288
S43	TX "cardiovascular disease*"	101,493
S42	MH "cardiovascular diseases+"	605,454
S41	S20 OR S21 OR S22 OR S23 OR S24 OR S25 OR S26 OR S27 OR S28 OR S29 OR S30 OR S31 OR S32 OR S33 OR S34 OR S35 OR S36 OR S37 OR S38 OR S39 OR S40	338,545
S40	TX "patient experience" OR TX "patient reported experience measures" OR TX "PREMs"	5,223
S39	(MH "consumer participation") OR TX "consumer participation" OR TX "patient participation"	21,858
S38	TX "patient centeredness"	704
S37	(MH "patient centered care") OR TX "patient-centered care"	33,394
S36	(MH "patient preference") OR TX "patient preference"	2,689
S35	TX "patient satisfaction"	64,477
S34	(MH "patient satisfaction+")	57,766
S33	TX "patient reported measure" OR "patient outcome assessment"	16,324
S32	TX "outcome measur*" OR "outcome metric*" OR "outcome indicator*" OR "outcome assessment"	135,925
S31	TX "patient-reported outcomes"	9,251
S30	MH "patient-reported outcomes+"	3,089
S29	MH "outcome assessment"	45,072
S28	TX "clinical measur*" OR "clinical metric*" OR "clinical indicator*" OR "clinical outcome*"	73,094
S27	MH "clinical indicators"	12,663
S26	TX "value measur*" OR "value metric*" OR "value indicator*"	275
S25	TX "process measur*" OR " process assessment" OR "process indicator*"	6,469
S24	MH "process assessment (health care)+"	8,217
S23	TX "performance measur*" OR "performance metric*" OR "performance rat*" OR "performance indicator*" OR "total performance score"	11,483
S22	TX "key performance indicators"	421
S21	MH "performance measurement systems"	2,270
S20	TX "quality measur*" OR "quality metric*" OR "quality rat*" OR "quality indicator*"	14,863
S19	S12 OR S13 OR S14 OR S15 OR S16 OR S17 OR S18	2,482,761
S18	TI ((cancer or oncology) N2 (center OR centers OR centre OR centres OR institute* OR facilit* OR "hospital unit*")) OR AB ((cancer or oncology) N2 (center OR centers OR centre OR centres OR institute* OR facilit* OR "hospital unit*"))	17,070
S17	(MH "Cancer Care Facilities")	5,712

S16	TI ((cardiac or heart or cardio*) N2 (center OR centers OR centre OR centres OR institute* OR facilit* OR "hospital unit*")) OR AB ((cardiac or heart or cardio*) N2 (center OR centers OR centre OR centres OR institute* OR facilit* OR "hospital unit*")) OR TI "coronary care unit*" OR AB "coronary care unit*"	4,158
S15	(MH "Academic Medical Centers")	75,682
S14	TX "center" OR "centers" OR "centre" OR "centres"	1,297,041
S13	TX "hospital" OR "hospitals"	1,627,654
S12	MH "Hospitals+"	120,141
S11	S1 OR S2 OR S3 OR S4 OR S5 OR S6 OR S7 OR S8 OR S9 OR S10	64,234
S10	TX "international consortium"	953
S9	TX "financial* incentive*"	2,916
S8	TX "financial* reward*"	370
S7	TI "payment" OR AB "payment"	13,802
S6	TX reimbursement	47,810
S5	MH "Reimbursement, Incentive"	2,669
S4	TX "pay for performance" OR P4P	1,908
S3	TX "value-driven"	506
S2	(MH "Value-Based Health Care") OR (MH "Value-Based Insurance") OR (MH "Value-Based Purchasing")	1,630
S1	TX value-based	5,827

((TX value-based) OR ((MH "Value-Based Health Care") OR (MH "Value-Based Insurance") OR (MH "Value-Based Purchasing")) OR (TX "value-driven") OR (TX "pay for performance" OR P4P) OR (MH "Reimbursement, Incentive") OR (TX reimbursement) OR (TI "payment" OR AB "payment") OR (TX "financial* reward*") OR (TX "international consortium") OR (TX "financial* incentive*")) AND ((MH "Hospitals+") OR (TX "hospital" OR "hospitals") OR (TX "center" OR "centers" OR "centre" OR "centres") OR ((MH "Academic Medical Centers")) OR (TI ((cardiac OR heart OR cardio*) N2 (center OR centers OR centre OR centres OR institute* OR facilit* OR "hospital unit*")) OR AB ((cardiac OR heart OR cardio*) N2 (center OR centers OR centre OR centres OR institute* OR facilit* OR "hospital unit*")) OR TI "coronary care unit*" OR AB "coronary care unit*") OR ((MH "Cancer Care Facilities")) OR (TI ((cancer OR oncology) N2 (center OR centers OR centre OR centres OR institute* OR facilit* OR "hospital unit*")) OR AB ((cancer OR oncology) N2 (center OR centers OR centre OR centres OR institute* OR facilit* OR "hospital unit*")))) AND ((TX "quality measur*" OR "quality metric*" OR "quality rat*" OR "quality indicator*") OR (MH "performance measurement systems") OR (TX "key performance indicators") OR (TX "performance measur*" OR "performance metric*" OR "performance rat*" OR "performance indicator*" OR "total performance score") OR (MH "process assessment (health care)+") OR (TX "process measur*" OR " process assessment" OR "process indicator*") OR (TX "value measur*" OR "value metric*" OR "value indicator*") OR (MH "clinical indicators") OR (TX "clinical measur*" OR "clinical metric*" OR "clinical indicator*" OR "clinical outcome*") OR (MH "outcome assessment") OR (MH "patient-reported outcomes+") OR (TX "patient-reported outcomes") OR (TX "outcome measur*" OR "outcome metric*" OR "outcome indicator*" OR "outcome assessment") OR (TX "patient reported measure" OR "patient outcome assessment") OR ((MH "patient satisfaction+")) OR (TX "patient satisfaction") OR ((MH "patient preference") OR TX "patient preference") OR ((MH "patient centered care") OR TX "patient-centered care") OR (TX "patient centeredness") OR ((MH "consumer participation") OR TX "consumer participation" OR TX "patient participation") OR (TX "patient experience" OR TX "patient reported experience measures" OR TX "PREMs")) AND ((MH "cardiovascular diseases+") OR (TX "cardiovascular disease*") OR (TI "cardi*" OR AB "cardi*" OR TI "heart" OR AB "heart") OR (MH "neoplasms+") OR (MH "oncology+") OR (TX "neoplasm*" OR "oncology" OR "cancer" "OR"))
