# Supplementary material for: Quality measurement for cardiovascular diseases and cancer in hospital value-based healthcare: a systematic review of the literature
Source: BMC Health Serv Res. 2022 Aug 1;22:979. doi: 10.1186/s12913-022-08347-x (PMC9341062; doi:10.1186/s12913-022-08347-x)
Supplement: Supplementary file 3 — Additional file 3. [file 12913_2022_8347_MOESM3_ESM.pdf]

Additional File	
**Article title:**	Quality Measurement for Cardiovascular Diseases and Cancer in Hospital Value-Based Healthcare: A Systematic Review of the Literature
**Author names:**	Rawia Abdalla, Milena Pavlova, Mohammed Hussein, and Wim Groot.
**Affiliation and e-mail address of the corresponding author:**	Maastricht University, Department of Health Services Research, CAPHRI, Maastricht University Medical Center, Faculty of Health, Medicine and Life Sciences, Maastricht, Limburg, The Netherlands.<br>r.abdalla@maastrichtuniversity.nl<br>Rawia85@yahoo.com
**Caption:**	This file includes the list of excluded studies.

Additional File 3

List of the excluded studies (n=25).

Studies excluded for having abstract only (n=17):

1. Bou Malham S, Njeim M, Ambulgekar N, et al. Does compliance with the heart failure inpatient quality measures prevent hospital readmission? Paper presented at: 34th Annual Meeting of the Society of General Internal Medicine; 2011; Phoenix, AZ. (Proposal ID # 12238)
2. Dobbs M, Heck S, Elkins K, Blackburn J, Boulanger B. Perfect care improves value for ischemic stroke patients, hospitals, and payors (S11.001). *Neurology*. 2015;84(14 Suppl):S11.001.
3. DeRosa P, Shenai M, Mancini B, Ecklund J, Cochran JW. Abstract WP289: Patient Satisfaction Survey Prior to Discharge in Neuroscience Unit: Evolving Gap in Hospital Operational Models and Patient Requests. *Stroke*. 2016;47(suppl_1):AWP289-AWP289. doi:doi:10.1161/str.47.suppl_1.wp289
4. White JRL, Palmer P, Sarma D, et al. Surgeon-Specific Performance on Breast Care Metrics: Results from a Large Integrated Cancer Network. Paper presented at: 72nd Annual Cancer Symposium of the Society of Surgical Oncology; Mar 27-30, 2019; California, SD. (Abstract P87)
5. Urick B, Stover A, Deal A, et al. Development and evaluation of patient-reported outcomes-based performance measures during chemotherapy. *J Clin Oncol*. 2020;38(15 suppl):e19175-e19175. doi:10.1200/JCO.2020.38.15_suppl.e19175.
6. Spinks T, Lee S, Shah K, Guzman A, Feeley T. A patient-centered outcome measurement approach for bundled payments in cancer care. *J Clin Oncol*. 2014;32(30 Suppl):266. doi:10.1200/jco.2014.32.30_suppl.266
7. Soliman R, Eweida W, Zamzam M, Elhaddad A, Abouelnaga S. The delivery of value-based healthcare for children with osteosarcoma at Children's Cancer Hospital Egypt. *J Clin Oncol.* 2017;35(15 Suppl):e18310-e18310. doi:10.1200/JCO.2017.35.15_suppl.e18310
8. Nguyen T, Peng J, O'Rourke C, et al. Patient ratings of hospital care using the HCAHPS survey following pancreatic surgery are influenced by pain management and not postoperative complications [abstract TP11-2]. *Ann Surg Oncol*. 2016;18(1 Suppl): S110.
9. Mooney K, Biber J, Hess R, Weeks H, Sweetenham J. Implementing routine assessment of patient-reported outcomes in cancer care. *J Clin Oncol*. 2017;35(8 Suppl):229. doi:10.1200/JCO.2017.35.8_suppl.229
10. McNiff K, Zhang Y, Fraile B, Hassett M. Deploying end-of-life utilization measures for oncology patients to assess hospital care quality and value. *J Clin Oncol*. 2018;36(30_suppl):106. doi:10.1200/JCO.2018.36.30_suppl.106
11. Maganty A, Yabes J, Bandari J, et al. An interdisciplinary claims-based quality algorithm for renal cancer for localized to life-limiting disease [abstract MP27-06]. *J Urol.* 2020;203(4 Suppl):e411-e412. doi:10.1097/JU.0000000000000866.06
12. Loo NM-M, Taddei T. Using a European Value-Based Medicine Approach to Evaluate Hepatocellular Carcinoma Care at a US Tertiary Care Center. *J Clin Oncol*. 2015;33(15 Suppl):e15118-e15118. doi:10.1200/jco.2015.33.15_suppl.e15118

13. Kreizenbeck K, Hughes E, Stewart F, et al. Regional initiative to define, collect, and report value metrics in cancer care. *J Clin Oncol*. 2014;32(30 Suppl):25. doi:10.1200/jco.2014.32.30_suppl.25
14. Johansen N, Perera S, Zeps N, Lippa J, Saunders C. Implementing a standardized breast cancer database in a private hospital in Western Australia – a pilot study (283). *Asia Pac J Clin Oncol*. 2016;12(S5 Suppl):66-168. doi:https://doi.org/10.1111/ajco.12646
15. Ives A, Millar L, Slavova-Azmanova N, et al. Measuring what's important to our patients: Continuous Improvement in Care-Cancer (CIC Cancer) Project. *Asia Pac J Clin Oncol*. 2018;14(Suppl):148.
16. Colman G, Hardeman E, Moorad J. Enhancing the quality of the patient experience in an academic medical center. *J Clin Oncol*. 2013;31(31 Suppl):149. doi:10.1200/jco.2013.31.31_suppl.149
17. Alvarnas J, Kassab T, Avanessian P, Pierce M, Levine A. The City of Hope (COH) performance excellence program (PEP): A physician incentive program to improve health care performance (HCP) in an academic medical center. *J Clin Oncol*. 2013;31(15 Suppl):e17556-e17556. doi:10.1200/jco.2013.31.15_suppl.e17556

Studies excluded as they are not in a value-based healthcare context (n=5):

18. Smith E, Saver J, Alexander D, et al. Clinical performance measures for adults hospitalized with acute ischemic stroke: performance measures for healthcare professionals from the American Heart Association/American Stroke Association. *Stroke*. 2014;45(11):3472-3498.
19. Shih T, Dimick J. Reliability of readmission rates as a hospital quality measure in cardiac surgery. *Ann Thorac Surg*. 2014;97(4):1214-1218.
20. McNair A, Whistance R, Forsythe R, et al. Core Outcomes for Colorectal Cancer Surgery: A Consensus Study. *PLoS Med*. 2016;13(8):e1002071.
21. Cohn D, Leitao M, Levenback C, et al. Reporting of quality measures in gynecologic oncology programs at Prospective Payment System (PPS)-Exempt Cancer Hospitals: an early glimpse into a challenging initiative. *Gynecol Oncol*. 2013;130(3):403-406.
22. Choi J, Shin D, Kang J, et al. Variations in process and outcome in inpatient palliative care services in Korea. *Support Care Cancer*. 2012;20(3):539-547.

Studies excluded as they are not specific for cardiovascular diseases or cancer (n=2):

23. Mulder J, Galema-Boers A, de Jong-Verweij L, Hazelzet J, Roeters van Lennep J. The development and first results of a health-related outcomes set in familial hypercholesterolemia (FH) patients: Knowledge is health. *Atherosclerosis*. 2020;293:11-17.
24. Kavalukas S, Baucom R, Geiger T, et al. Benchmarking patient satisfaction scores in a colorectal patient population. *Surg Endosc*. 2021;35(1):309-316.

Studies that focus on outcomes' selection characteristics (n=1):

25. Moloney R, Messner D, Wessler Z, Tunis S. Proposed framework for patient-centered outcomes-based measures in alternative payment models. *Am J Manag Care*. 2021;27(2):80-84. doi:10.37765/ajmc.2021.88586
