# Supplementary material for: Quality measurement for cardiovascular diseases and cancer in hospital value-based healthcare: a systematic review of the literature
Source: BMC Health Serv Res. 2022 Aug 1;22:979. doi: 10.1186/s12913-022-08347-x (PMC9341062; doi:10.1186/s12913-022-08347-x)
Supplement: Supplementary file 4 — Additional file 4. [file 12913_2022_8347_MOESM4_ESM.pdf]

Additional File	
**Article title:**	Quality Measurement for Cardiovascular Diseases and Cancer in Hospital Value-Based Healthcare: A Systematic Review of the Literature
**Author names:**	Rawia Abdalla, Milena Pavlova, Mohammed Hussein, and Wim Groot.
**Affiliation and e-mail address of the corresponding author:**	Maastricht University, Department of Health Services Research, CAPHRI, Maastricht University Medical Center, Faculty of Health, Medicine and Life Sciences, Maastricht, Limburg, The Netherlands. r.abdalla@maastrichtuniversity.nl Rawia85@yahoo.com
**Caption:**	This file includes a summary of the included studies.

Additional File 4
Table 1: Summary of the included studies (n=37).

Author, Year, Country	Objectives	Design	Conclusion/ Recommendations	Outcome Selection Criteria	Methodological Quality
**Zack [38] 2019,**<br>**International Consortium for Health Outcomes Measurement (ICHOM)**	Define a standard set of patient-centered outcomes for evaluating hypertension management in low- and middle-income countries.	Consensus group	A core list of 18 outcomes for evaluating hypertension care was presented along with 12 case-mix variables. They account for the unique challenges healthcare providers and patients face in low- and middle-income countries, yet are relevant to all settings. It is believed that it is a vital step toward international benchmarking in hypertension care and, ultimately, value-based hypertension management.	▪ Importance to patients with hypertension<br>▪ Ease of measuring<br>▪ Modifiable with quality improvement efforts.	Good
**Veghel [39] 2016, Netherlands**	Assess patient-relevant outcomes of delivered cardiovascular care by focusing on disease management as determined by a multidisciplinary heart team, to establish and share best practices by comparing outcomes, and to embed value-based decision-making to improve quality and efficiency in Dutch heart centers.	Prospective cohort	Consensus has been created on a small set of 9 outcome measures, case-mix variables, and initial conditions. It was concluded that transparent publication of outcomes drives the improvement of quality within heart centers. Transparent communication on outcomes is feasible, safe and cost-effective, and stimulates professional decision-making and disease management.	▪ Impact on quality of life of patients<br>▪ Prevalence of the outcome<br>▪ Possibility for physicians to influence the outcome<br>▪ Feasibility of data collection<br>▪ Quality of the definition	Good
**Shashikumar [40] 2020, USA**	Investigate outcomes of frail patients with heart failure using a claims-based frailty index.	Retrospective cohort	Frail patients with heart failure had significantly poorer outcomes than non-frail patients after accounting for comorbidities. Clinicians should screen for frailty to identify high-risk patients who could benefit from targeted intervention. Policymakers should perform risk adjustments for frailty for more equitable quality measurement and financial incentive allocation.	Not reported in the study	Fair
**Salinas [41] 2016,**<br>**ICHOM**	Define an international standard set of patient centered stroke outcome measures for use in a variety of healthcare settings in order to enable assessment of healthcare value in stroke management.	Consensus group	A consensus stroke measure Standard Set was developed as a simple, pragmatic method to increase the value of stroke care. The set should be validated in practice when used for monitoring and comparisons across different care settings.	▪ Pragmatism over idealism<br>▪ Completeness in data collection<br>▪ Measures that can be collected by retrospective abstraction<br>▪ Instruments that are perpetually freely available & ideally with a digital platform<br>▪ Instruments made of modular subunits that permit recombination of elements<br>▪ Measures robust to comparison in both low- and high-income countries & with available cost-utility values.	Fair

Author, Year, Country	Objectives	Design	Conclusion/ Recommendations	Outcome Selection Criteria	Methodological Quality
**Rimmele [42] 2020, Germany**	Assess the self-reported health-related quality of life with the International Consortium for Health Outcome Measurement - Standard Set for Stroke (ICHOM-SSS) in routine clinical practice and to identify predictors of impaired quality of life in unselected patients treated for acute stroke.	Prospective exploratory observational (cohort)	Patient-reported health status was assessed by the Patient-Reported Outcomes Measurement Information System (PROMIS-10) questionnaire as part of the ICHOM-SSS. Integrated into the routine of acute stroke care, systematic assessment of patient-reported outcomes reveals impairments in physical and mental health. The main predictors are the severity of stroke symptoms and comorbidities such as hypertension and diabetes.	Not reported in the study	Good
**Osnabrugge [43] 2014, USA**	Define value in Coronary Artery Bypass Grafting (CABG) and provide a framework to identify high-value centers.	Prospective cohort	Risk-adjusted length of stay and risk-adjusted combined morbidity/mortality are important outcome measures for assessing value in cardiac surgery. The proposed framework (combining risk-adjusted quality and risk-adjusted resource use measures) can be used to define value in CABG and identify high-value centers, thereby providing information for quality improvement and pay-for-performance initiatives.	Not reported in the study	Good
**Neilson [44] 2019, USA**	Identify outcome measures that are meaningful to key stakeholders to inform value-based contracts for coronary artery disease medications.	Consensus group	Eleven outcomes reached a consensus for importance. ‘‘Preventing heart attacks’’ was selected as the most meaningful outcome, while ‘‘preventing death’’ ranked second. The results of the study verify the utility of a widely used clinical coronary artery disease outcome measure, myocardial infarction events, for the purpose of pharmaceutical value-based contracting.	Not reported in the study	Good
**Kinnier [45] 2016, USA**	(1) Develop a novel measure of Venous Thromboembolism (VTE) prophylaxis that requires early ambulation, mechanical prophylaxis, and chemoprophylaxis throughout the hospitalization, and (2) compare hospital performance on the Surgical Care Improvement Project process measure to this novel measure	Comparative cross-sectional	This new measure incorporates the critical aspects of VTE prevention to ensure defect-free care. After additional evaluation, this composite VTE prophylaxis measure with appropriate exclusion criteria may be a better alternative to the existing VTE process and outcome measures that is subject to surveillance bias.	Not reported in the study	Fair
**Ju [46] 2014, USA**	Assess the presence and extent of VTE surveillance bias using high-quality clinical data.	Retrospective cohort	Even when examined with clinically ascertained outcomes and detailed risk adjustment, VTE rates reflect hospital imaging use and perhaps signify vigilant, high-quality care. The VTE outcome measure may not be an accurate quality indicator and should likely not be used in public reporting or pay-for-performance programs.	Not reported in the study	Fair

Author, Year, Country	Objectives	Design	Conclusion/ Recommendations	Outcome Selection Criteria	Methodological Quality
**Fonarow [47] 2012, USA**	Evaluate the degree to which hospital outcome ratings and potential eligibility for financial incentives are altered after including initial stroke severity in a claims-based risk model for hospital 30-day mortality for acute ischemic stroke.	Retrospective cohort	Adding stroke severity as measured by the National Institutes of Health Stroke Scale to a hospital 30-day risk model based on claims data for Medicare beneficiaries with acute ischemic stroke was associated with considerably improved model discrimination and change in mortality performance rankings for a substantial portion of hospitals. These findings suggest that it may be critical to collect and include stroke severity for optimal hospital risk adjustment of 30- day mortality for Medicare beneficiaries with acute ischemic stroke.	Not reported in the study	Fair
**MORSS [48] 2016, USA**	Evaluate associations between heart failure patient perspectives of care and publicly reported processes and outcomes.	Retrospective cross-sectional	Publicly reported process quality measures were no longer associated with outcomes, but higher patient perspectives of care were associated with lower heart failure readmissions. These associations support continued reevaluation of these measures and increased emphasis on patient experience and outcomes, as planned for value-based purchasing.	Not reported in the study	Fair
**Dharmarajan [49] 2017, USA**	Examine the relationship between hospital 30- day risk-standardized mortality rates for older patients (aged ≥65 years) and those for younger patients (aged 18 to 64 years) and all patients (aged ≥18 years) with acute myocardial infarction.	Retrospective cohort	Hospital mortality rankings for older patients with acute myocardial infarction inconsistently reflect rankings for younger patients. The incorporation of younger patients into the assessment of hospital outcomes would permit a further examination of the presence and effect of age-related quality differences.	Not reported in the study	Fair
**Daeter [50] 2018, Netherlands**	Share the standard set of outcome measures as developed by Meetbaar Beter Initiative, and show how the standard set is presented and published to support the improvement of cardiac care.	Consensus group	Meetbaar Beter has defined, implemented, and validated a comprehensive set of 15 patient-relevant outcome measures for coronary artery disease. The variation of the results among the centers indicates that there are sufficient opportunities to further improve cardiac care in the Netherlands.	▪ Patient relevance<br>▪ Medical relevance<br>▪ Patient volume	Fair
**Curcio [51] 2021, USA**	Analyze quality of life at baseline, one month, and one year following cardiac surgery as a primary outcome in a non-emergent, all comers in the cardiac surgery population	Prospective cohort	Quality of life and other patient-centered outcomes are improved at one month and continue to improve throughout the year. Knowledge of these data is important for patient selection, fully informed consent, and shared decision-making.	Not reported in the study	Good
**Blay [52] 2019, USA**	Examine how hospital Patient Safety Indicator (PSI) 90 scores would change if the VTE measure was removed from the calculation of this composite measure.	Cross-sectional	Inclusion of the surveillance bias-prone VTE outcome measure in the PSI 90 composite disproportionately penalizes larger, academic hospitals and those that care for sicker patients. Removal of the VTE outcome measure from PSI 90 should be strongly considered.	Not reported in the study	Fair

Author, Year, Country	Objectives	Design	Conclusion/ Recommendations	Outcome Selection Criteria	Methodological Quality
**Bilimoria [53] 2013, USA**	Examine whether a surveillance bias influences the validity of reported VTE rates.	Retrospective cohort	Hospitals With higher quality scores had higher VTE prophylaxis rates but worse risk-adjusted VTE rates. Increased hospital VTE event rates were associated with increasing hospital VTE imaging use rates. Surveillance bias limits the usefulness of the VTE quality measure for hospitals working to improve quality and patients seeking to identify a high-quality hospital.	Not reported in the study	Good
**Akmaz [54] 2019, Netherlands**	Present process measures that impact on outcome measures for surgical aortic valve replacement and transcatheter aortic valve replacement within value-based healthcare.	Qualitative explorative case study (semi-structured interviews and focus group)	This study proposes an addition of 12 process measures to standard sets of outcome measures to improve healthcare quality. It illustrates a clear method for identifying process measures with an impact on health outcomes in the future.	Not reported in the study	Good
**McNamara [55] 2015, ICHOM**	Define a consensus standard set of outcome measures and risk factors for tracking, comparing, and improving the outcomes of coronary artery disease care.	Consensus group	The ICHOM developed and recommended this set of 13 specific outcomes and other patient information to be measured for all patients with coronary artery disease.	▪ Frequency of the outcome<br>▪ Impact on the patient<br>▪ Potential to modify the outcome<br>▪ Feasibility of “capturing” the outcome in clinical practice.<br>▪ risk-adjustment variables were selected based on their relevance, independence, and feasibility of measurement.	Fair
**Burns [56] 2020, ICHOM**	Define a standard pragmatic patient-centered outcome set for heart failure to improve patient care and permit comparison across regions and health care systems. The set is intended to be both a management and a research tool.	Consensus group	The ICHOM developed a set of 17 outcomes designed to capture, compare, and improve care for heart failure, with feasibility and relevance for patients and clinicians worldwide.	▪ Frequency of occurrence for the outcome of interest<br>▪ Impact of a change in the outcome on the patient<br>▪ Outcome modification potential<br>▪ Feasibility of data collection at the primary care level<br>▪ How meaningful the outcome is as reported by survey or focus group patients<br>▪ Cost to patients or systems.	Good
**Zerillo [57] 2017, ICHOM**	Define an international standard set of patient-centered outcome measures for colorectal cancer by the ICHOM.	Consensus group	A standardized set of 31 patient-centered outcome measures to inform value-based health care in colorectal cancer was developed. Pilot efforts are underway to measure the standard set among members of the working group.	Not reported in the study	Good

Author, Year, Country	Objectives	Design	Conclusion/ Recommendations	Outcome Selection Criteria	Methodological Quality
**Wang [58] 2016, Tawian**	Examine the association of outcomes and medical expenditures with a bundled-payment pay-for-performance program for breast cancer in Taiwan compared with a fee-for-service program.	Case-control	In Taiwan, compared with the regular fee-for-service program, bundled payment may lead to better adherence to quality indicators, better outcomes (i.e., survival rates), and more effective cost control over time.	Not reported in the study	Good
**Egdom [59] 2019, Netherlands**	Describe the development of breast cancer outcomes set, data integration within Electronic Health Records (EHR), and clinical use.	Mixed method (Consensus group and cross-sectional)	A standard outcome set was developed for breast cancer. Assessment of patient-reported as well as provider-reported outcomes was implemented within the standard of breast cancer care. For this, dedicated resources, change of culture and practice, and improved knowledge and awareness about Value-based healthcare were essential. A secure electronic platform, directly linked to the EHR, was designed to measure PROMs during the outpatient phase. Patients reacted positively to the use of PROMs in daily clinical cancer care.	Not reported in the study	Good
**Stover [60] 2020, USA**	Elicit stakeholder recommendations for Patient-Reported Outcome - Performance Measures (PRO-PMs) that assess how patients feel and function, and conduct feasibility testing at six cancer centers.	Mixed method (Consensus group and cross-sectional)	Clinicians, patients, and other stakeholders agree that performance measures that are based on how patients feel and function would be an important addition to quality measurement. This study also shows that PRO-PMs can be feasibly captured at home during systemic therapy and are acceptable to patients. PRO-PMs may add value to the portfolio of performance measures as oncology transitions from fee-for-service payment models to performance-based care that emphasizes outcome measures.	Not reported in the study	Good
**Sohn [61] 2016, USA**	Determine whether adherence to any of the available payer-driven quality measures influences patient-centered outcomes, including health-related quality of life, patient satisfaction, and treatment-related complications.	Prospective cohort	Compliance with available nationally endorsed quality indicators, which were designed to incentivize effective and efficient care, was not associated with clinically important changes in patient-centered outcomes (health-related quality of life), satisfaction, or complications within 1-year. This represents an opportunity to identify alternative measures that may influence patient-centered outcomes.	Not reported in the study	Good
**Nguyen [62] 2018, Belgium**	Measure outcomes that matter to patients with advanced lung cancer treated with concurrent chemoradiation beyond survival in general clinical practice.	Prospective cohort	The assault on health-related quality of life during concurrent chemoradiation for locally advanced lung cancer is considerable. Loss of physical and role functioning persists up to 6 and 9 months after therapy ends, respectively. Measuring patient-reported outcomes can help to identify issues for improvement of the value of care delivered.	Not reported in the study	Fair

Author, Year, Country	Objectives	Design	Conclusion/ Recommendations	Outcome Selection Criteria	Methodological Quality
**Martin [63] 2015, ICHOM**	Develop a standard set of multidimensional patient-centered health outcomes for tracking, comparing, and improving localized prostate cancer treatment value.	Consensus group	A simple, easily implemented set of outcomes was defined, and it is believed that it should be measured in all men with localized prostate cancer as a crucial first step in improving the value of care.	Not reported in the study	Fair
**Lobatto [64] 2019, Netherlands**	Describe Value-Based Healthcare (VBHC) perioperative outcomes for patients with pituitary tumors up to 6 months postoperatively	Prospective cohort	Though challenging, outcomes of surgical intervention for patients with pituitary tumors can be reflected through a VBHC-based comprehensive outcome set that can distinguish outcomes among different patient groups with respect to tumor type.	Not reported in the study	Fair
**Liang [65] 2015, USA**	Evaluate compliance with 8 quality indicators proposed by the Society of Gynecologic Oncology for ovarian cancer.	Cross-sectional	Compliance with strict definitions of ovarian cancer quality indicators varies depending on the care delivered and documentation of that care. Increased attention to comprehensive surgical staging and timely initiation of chemotherapy appears warranted.	Not reported in the study	Fair
**Landercasper [66] 2018, USA**	Report member performance on 9 breast cancer quality measures that were selected by the American Society of Breast Surgeons (ASBrS) for the Centers for Medicare and Medicaid Services (CMS) Quality Payment Programs (QPP) and other performance improvement programs.	Cross-sectional	Surgeons self-reported a large number of specialty-specific patient-measure encounters into a registry for self-assessment and participation in QPP. Despite high levels of performance demonstrated initially in 2011 with minimal subsequent change, the ASBrS concluded ‘‘perfect’’ performance was not a realistic goal for QPP. Thus, after a review of our normative performance data, the ASBrS recommended different benchmarks than CMS for each quality measure.	Not reported in the study	Poor
**Fayanju [67] 2016, USA**	Describe the process of developing value-based measures for breast cancer patients, and the dynamic capture of these metrics via a new electronic health record.	Consensus group	A set of 22 patient-centered outcome measures for breast cancer was developed. The integration of patient-centered outcomes—including outcomes directly reported by patients themselves—into the electronic health record will facilitate the transparent reporting of outcomes as well as interfacility benchmarking, both of which are becoming important features of the healthcare environment.	Not reported in the study	Good
**Cramer [68] 2021, Netherlands**	Establish a relevant set of outcome indicators for lung cancer.	Mixed method (consensus group and cohort)	A relevant set of outcome indicators for lung cancer was systematically developed. This set has the potential to compare the quality of care between hospitals and inform patients with lung cancer about outcomes.	▪ Relevance to the patient<br>▪ Medical relevance<br>▪ Relevance for patient populations,	Fair

Author, Year, Country	Objectives	Design	Conclusion/ Recommendations	Outcome Selection Criteria	Methodological Quality
**Morgans [69] 2015, ICHOM**	Develop a standard set of outcomes relevant to men with advanced prostate cancer to follow during routine clinical care.	Consensus group	A standardized set of patient-centered outcomes to be followed during routine care for all men with advanced prostate cancer was developed. The international multidisciplinary group identified clinical data and PROMs that serve as a basis for international health outcome comparisons and quality- of care assessments.	▪ Scope and outcome domains<br>▪ Outcome definitions and measures<br>▪ Case-mix factors<br>▪ Case-mix measures.	Fair
**Mak [70] 2016, ICHOM**	Define a recommended standard set of outcomes and corresponding baseline demographic, clinical and tumor characteristics (case-mix variables) for patients with lung cancer.	Consensus group	An international consensus outcome set of the most important outcomes for lung cancer patients, along with relevant case-mix variables was developed. Work is ongoing to support the adoption and reporting of these measures globally.	Not reported in the study	Fair
**Lagendijk [71] 2018, Netherlands**	Collect patient-reported outcomes retrospectively and analyze differences per type of surgery delivered. The aim was to obtain reference values helpful in shared decision-making.	Retrospective cross-sectional	Patient-reported outcome scores were associated with age, time since surgery, type of surgery, and radiation therapy in breast cancer patients. The scores serve as a reference value for different types of surgery in the study population and enable prospective use of patient-reported outcomes in shared decision-making.	Not reported in the study	Fair
**Javid [72] 2017, USA**	Better understand what health-related quality of life domains and processes of care define high-quality surgical care for women undergoing mastectomy for breast cancer from both the patient and clinician perspective.	Exploratory qualitative study	Patients and clinicians largely agreed on important health-related quality of life domains, including emotional well-being, education, communication, and process of care. The stakeholder advisory panel recommended that the BREAST-Q is an ideal tool to begin developing novel quality improvement benchmarks focused on patient-reported outcomes.	Not reported in the study	Good
**Escudero [73] 2020, Spain**	Adapt the standard set of variables for newly diagnosed lung cancer patients developed by the ICHOM to the Spanish setting in order to facilitate the use of this standard set.	Consensus group	The adaptation of ICHOM standard set to the Spanish setting paves the way to standardize the collection of variables in lung cancer and promote the incorporation of patients' perspectives in lung cancer management. The variables agreed upon included outcomes and case-mix variables.	Not reported in the study	Good
**Ong [74] 2017, ICHOM**	Develop a standard set of value-based patient-centered outcomes for breast cancer	Consensus group	A set of 26 patient-centered outcome measures for breast cancer was developed along with case-mix variables. This set is deemed to be most important to patients with breast cancer, and generally applicable worldwide. It is recommended that the set is collected in routine clinical practice.	▪ Outcomes coverage<br>▪ psychometric quality<br>▪ Clinical interpretability<br>▪ Feasibility of implementation in daily practice	Good

Abbreviations: ASBrS indicates American Society of Breast Surgeons; CABG, Coronary Artery Bypass Grafting; CMS, Centers for Medicare and Medicaid Services; HER, Electronic Health Records; ICHOM, International Consortium for Health Outcomes Measurement; ICHOM-SSS, International Consortium for Health Outcome Measurement - Standard Set for Stroke; PROMs, Patient Reported Outcome Measures; PROMIS-10, Patient-Reported Outcomes Measurement Information System; PRO-PMs, Patient-Reported Outcome -Performance Measures; PSI, Patient Safety Indicator; QPP, Quality Payment Programs; VBHC, Value-Based HealthCare; VTE, Venous Thromboemoblism.
