# Supplementary material for: Quality measurement for cardiovascular diseases and cancer in hospital value-based healthcare: a systematic review of the literature
Source: BMC Health Serv Res. 2022 Aug 1;22:979. doi: 10.1186/s12913-022-08347-x (PMC9341062; doi:10.1186/s12913-022-08347-x)
Supplement: Supplementary file 5 — Additional file 5. [file 12913_2022_8347_MOESM5_ESM.pdf]

Additional File	
**Article title:**	Quality Measurement for Cardiovascular Diseases and Cancer in Hospital Value-Based Healthcare: A Systematic Review of the Literature
**Author names:**	Rawia Abdalla, Milena Pavlova, Mohammed Hussein, and Wim Groot.
**Affiliation and e-mail address of the corresponding author:**	Maastricht University, Department of Health Services Research, CAPHRI, Maastricht University Medical Center, Faculty of Health, Medicine and Life Sciences, Maastricht, Limburg, The Netherlands.<br>r.abdalla@maastrichtuniversity.nl<br>Rawia85@yahoo.com
**Caption:**	This file includes a summary of quality measures reported in the studies.

Additional File 5
Table 2: Summary of quality measures reported in the studies

Author, Year	Condition/ Disease	Survival	Degree of Health or Recovery	Time to recovery & time to return to normal activities	Disutility of care or treatment process	Sustainability of health or recovery	Long-term consequences of therapy	Process Measures
**Zack [38] 2019**	Hypertentoin	▪ Overall survival and cardiovascular survival † [or] ‡	▪ QoL § [a,b,c or d]<br>▪ Blood pressure control (< 140/90 mm Hg) [‡]<br>▪ Erectile dysfunction §<br>▪ Patient satisfaction §<br>▪ Health beliefs § [e]<br>▪ Medication adherence § [f]<br>▪ Pill burden §	▪ Access to care §<br>▪ Access to medication §	▪ Adverse events and side effects of medication ‡	▪ Ischemic heart disease ‡<br>▪ Cerebrovascular disease ‡<br>▪ Atrial fibrillation ‡<br>▪ Heart failure ‡<br>▪ Peripheral artery disease ‡<br>▪ Chronic renal disease ‡<br>▪ Hypertensive urgency or hypertensive emergency ‡	Not reported in the study	Not reported in the study
**Veghel [39] 2016**	Cardiovascular	▪ Risk-adjusted 120-day mortality for all so-called consolidated AVD ‡<br>▪ Risk-adjusted 120-day mortality after CABG ‡<br>▪ Risk-adjusted 1-year mortality after PCI ‡<br>▪ Risk-adjusted long-term survival rates after AVR ‡	▪ QoL § [c or g]	Not reported in the study	▪ Risk-adjusted implantations of a new permanent pacemaker within 30 days after TAVI ‡	▪ Risk-adjusted readmission due to myocardial infarction within 30-day for consolidated CAD‡<br>▪ Risk-adjusted target vessel revascularization rate within 1 year of PCI ‡	Not reported in the study	Not reported in the study
**Salinas [41] 2016**	Stroke	▪ All-cause mortality †	▪ QoL § [b]<br>▪ Mobility; Self-care & grooming; Feeding; Ability to communicate §<br>▪ Adherence to smoking cessation advice §<br>▪ Ability to return to usual activities ‡ [&] § [c or h]	Not reported in the study	▪ Acute complications (Symptomatic intracerebral hemorrhage after thrombolysis) ‡	▪ Recurrence of disease (stroke or Transient Ischemic Attack) ‡ [or] §	Not reported in the study	Not reported in the study

Author, Year	Condition/ Disease	Survival	Degree of Health or Recovery	Time to recovery & time to return to normal activities	Disutility of care or treatment process	Sustainability of health or recovery	Long-term consequences of therapy	Process Measures
**Rimmele [42] 2020**	Stroke	Not reported in the study	QoL § b & i	Not reported in the study	Not reported in the study	Not reported in the study	Not reported in the study	Not reported in the study
**Osnabrugge [43] 2014**	Coronary Artery Bypass Grafting (CABG)	Risk-adjusted morbidity/ mortality ‡	Not reported in the study	Not reported in the study	Risk-adjusted length of stay ‡	Not reported in the study	Not reported in the study	Not reported in the study
**Neilson [44] 2019**	Coronary Artery Disease	Not reported in the study	▪ Improving QoL §<br>▪ Improving function §<br>▪ Reducing shortness of breath §<br>▪ Reducing chest pain §<br>▪ Reducing fatigue §	Not reported in the study	▪ Preventing heart attacks ‡<br>▪ Preventing need for a repeat heart procedure ‡<br>▪ Preventing death ‡<br>▪ Preventing stroke or mini-stroke ‡<br>▪ Preventing heart failure from heart disease ‡<br>▪ Reducing medication complications like major bleeding ‡<br>▪ Preventing stent blockage ‡<br>▪ Preventing heart medication switches because of failure of treatment, side effects or other issues with medications ‡	Not reported in the study	Not reported in the study	Not reported in the study
**Kinnier [45] 2016**	Venous Thromboembolism (VTE)	Not reported in the study	Not reported in the study	Not reported in the study	▪ VTE Rate ‡	Not reported in the study	Not reported in the study	A composite VTE prophylaxis measure that requires daily ambulation, mechanical prophylaxis using continuous sequential compression devices, and properly timed and dosed chemoprophylaxis throughout the hospitalization ‡

Author, Year	Condition/ Disease	Survival	Degree of Health or Recovery	Time to recovery & time to return to normal activities	Disutility of care or treatment process	Sustainability of health or recovery	Long-term consequences of therapy	Process Measures
**MORSS [48] 2016**	Heart failure	▪ 30-day heart failure mortality ‡	▪ Patient experience using both the Hospital Consumer Assessment of Healthcare Providers and Systems (HCAHPS) and Press Ganey Inpatient surveys §	Not reported in the study	Not reported in the study	▪ 30-day heart failure readmissions ‡	Not reported in the study	▪ Heart Failure Patients Given Discharge Instructions ‡<br>▪ Heart Failure Patients Given an Evaluation of Left Ventricular Systolic Function ‡<br>▪ Heart Failure Patients Given ACE Inhibitor or ARB for Left Ventricular Systolic Dysfunction ‡
**Daeter [50] 2018**	Coronary Artery Disease	▪ Long-term survival (≤5 years) for CABG, PCI, and CT †<br>▪ 1-year mortality for CABG, PCI, and CT †<br>▪ 120-day mortality for CABG †<br>▪ 30-day mortality for PCI †	▪ QoL § [c or g]<br>▪ Chest pain for CT §	Not reported in the study	▪ Surgical reexploration (≤30 days) for CABG ‡<br>▪ Cerebrovascular accident (≤72 hours) for CABG ‡<br>▪ Deep sternal wound infection (≤30 days) for CABG ‡<br>▪ Urgent CABG (≤24 hours) for PCI ‡	▪ Myocardial infarction (≤30 days) for CABG, PCI, and CT ‡<br>▪ Occurrence of target vessel revascularization (≤1 year) for PCI ‡<br>▪ Free of myocardial infarction for CABG ‡<br>▪ Free of coronary artery reintervention for CABG ‡<br>▪ Free of Major Adverse Cardiac Event for CT ‡	Not reported in the study	Not reported in the study
**Curcio [51] 2021**	Cardiac surgery	Not reported in the study	▪ QoL § [j]	Not reported in the study	Not reported in the study	Not reported in the study	Not reported in the study	Not reported in the study
**Akmaz [54] 2019**	Surgical Aortic Valve Replacement (SAVR) and Transcatheter Aortic Valve	▪ 120-day mortality<br>▪ Long-term survival ≤ 1 year)<br>▪ Procedural mortality for TAVR	▪ QoL §<br>▪ Mobilization<br>▪ New York Heart Association (NYHA) classification for TAVR	Not reported in the study	▪ Cerebrovascular Accident<br>▪ Deep sternal wound Infection for SAVR	▪ Freedom of valve reintervention	Not reported in the study	▪ Number of times that deficient information provision to SAVR patients causes negative outcomes

Author, Year	Condition/ Disease	Survival	Degree of Health or Recovery	Time to recovery & time to return to normal activities	Disutility of care or treatment process	Sustainability of health or recovery	Long-term consequences of therapy	Process Measures
	Replacement (TAVR)	▪ 30-day mortality for TAVR			▪ Implantation of new permanent pacemaker<br>▪ Vascular complications for TAVR<br>▪ Infections for SAVR including lung infections<br>▪ Re-sternotomy for SAVR			▪ Type of SAVR / TAVR prosthesis<br>▪ Brand of TAVR prosthesis<br>▪ Number of times the frailty score of TAVR/SAVR patient >75 years is measured<br>▪ Time between TAVR/SAVR surgery indication and surgery<br>▪ Number of times that anticoagulants are stopped within 3 days before surgery<br>▪ Time in hours between TAVR/SAVR surgery and permanent pacemaker implantation<br>▪ Percentage of standardized pain measurements
**McNamara [55] 2015**	Coronary Artery Disease	▪ All-cause mortality for All patients with coronary artery disease †<br>▪ Mortality post-procedure and place of death (PCI and CABG) † or ‡	▪ QoL § [k & l]<br>▪ Angina § [m]<br>▪ Dyspnea § [n]<br>▪ Depression § [o]<br>▪ Functional status §	Not reported in the study	▪ Admissions (for acute myocardial infarction, hemorrhagic stroke, ischemic stroke or heart failure) †<br>▪ Procedural interventions (PCI and /or CABG)†<br>▪ Acute renal failure†<br>▪ Acute complications of treatment (PCI & CABG) † or ‡	Not reported in the study	Not reported in the study	Not reported in the study

Author, Year	Condition/ Disease	Survival	Degree of Health or Recovery	Time to recovery & time to return to normal activities	Disutility of care or treatment process	Sustainability of health or recovery	Long-term consequences of therapy	Process Measures
					▪ Major surgery complications (CABG only) ‡<br>▪ Interventional cardiology complications (PCI only) ‡			
**Burns [56] 2020**	Heart Failure	Mortality †	▪ QoL § [b, o & j]<br>▪ Physical activity § [p]<br>▪ Financial burden §	Not reported in the study	▪ Medication side effects ‡<br>▪ Complications of treatment ‡ [or] §<br>▪ Number of hospital appointments †<br>▪ Number of hospital readmissions †<br>▪ Length of stay †	Not reported in the study	Not reported in the study	Not reported in the study
**Zerillo [57] 2017**	Colorectal Cancer (CRC)	▪ Overall survival †<br>▪ Cause of death (for all patients) †<br>▪ Recurrence-free survival (for Patients with curative intent ‡<br>▪ Progression-free survival (for Patients with advanced disease‡	▪ QoL § [q,r, s & t]<br>▪ Pathological or clinical complete response (for patients with rectal cancer receiving neoadjuvant therapy ‡<br>▪ Margin status (for patients with rectal cancer receiving surgery) ‡<br>▪ Quality of death for patients with advanced disease † [&] ‡	Not reported in the study	▪ Short-term complications of treatment ‡	Not reported in the study	Not reported in the study	Not reported in the study
**Wang [58] 2016**	Breast Cancer	▪ Event-free survival †<br>▪ Overall survival rates †	Not reported in the study	Not reported in the study	Not reported in the study	Not reported in the study	Not reported in the study	▪ Cytologic or histologic confirmation before definitive surgery †<br>▪ Patients with ductal carcinoma in situ do not undergo axillary node dissection †<br>▪ Axillary node dissection with ≥10

Author, Year	Condition/ Disease	Survival	Degree of Health or Recovery	Time to recovery & time to return to normal activities	Disutility of care or treatment process	Sustainability of health or recovery	Long-term consequences of therapy	Process Measures
								axillary lymph nodes for patients with invasive cancer †<br>▪ Radiotherapy for invasive cancer after breast conservation surgery †<br>▪ Tumor size recorded †<br>▪ Adjuvant chemotherapy for patients 50 y or younger with positive axillary lymph nodes †<br>▪ Adjuvant chemotherapy or hormone therapy for patients older than 50 y with positive axillary lymph nodes †
**Egdom [59] 2019**	Breast Cancer	▪ Mortality ‡<br>▪ Recurrence Free Survival ‡	▪ QoL § [u,q,s,v,w,x & y]<br>▪ QoL of carers [z]	Not reported in the study	▪ Complications using Clavien-Dindo classification system ‡	▪ Number of reoperations due to positive margins ‡	Not reported in the study	Not reported in the study
**Stover [60] 2020**	Cancer	Not reported in the study	▪ QoL § (Toxicities; Symptom control; emotional distress; financial burden; Physical function; Patients' preferences for symptom management; Pain)<br>▪ Care experiences and satisfaction with care §	Not reported in the study	Not reported in the study	Not reported in the study	Not reported in the study	▪ Care coordination<br>▪ Information provision that is understandable<br>▪ Communication<br>▪ Adherence to guideline-concordant care
**Sohn [61] 2016**	Prostate cancer	Not reported in the study	▪ QoL § [aa]<br>▪ Patient satisfaction § [bb]	Not reported in the study	▪ Treatment-related complications ‡	Not reported in the study	Not reported in the study	▪ Avoidance of overuse of bone scan in men with low-risk tumors<br>▪ Androgen-Deprivation Therapy

Author, Year	Condition/ Disease	Survival	Degree of Health or Recovery	Time to recovery & time to return to normal activities	Disutility of care or treatment process	Sustainability of health or recovery	Long-term consequences of therapy	Process Measures
								for high-risk patients undergoing radiation<br>▪ Documentation cT stage, biopsy Gleason in newly diagnosed<br>▪ Documentation of digital rectal examination, cT stage, biopsy Gleason before primary therapy<br>▪ Documentation of discussion of treatment options<br>▪ Documentation of pathologic T and N stage, Gleason score, and margin status on pathology report in men undergoing radical prostatectomy
**Nguyen [62] 2018**	Lung cancer	▪ Survival ‡<br>▪ Treatment-related Death ‡	▪ QoL § [q]<br>▪ Quality of death ‡	Not reported in the study	Not reported in the study	Not reported in the study	Not reported in the study	Not reported in the study
**Martin [63] 2015**	Prostate Cancer	▪ Overall survival †<br>▪ Cause-specific survival †<br>▪ Metastasis-free survival ‡ [or] §<br>▪ Biochemical recurrence-free survival ‡ [or] §	▪ QoL § [aa]	Not reported in the study	▪ Acute complications for surgery patients using Clavien-Dindo classification ‡ [or] §<br>▪ Acute complications for radiation patients using the Common Terminology Criteria for Adverse Events ‡ [or] §	Not reported in the study	Not reported in the study	Not reported in the study
**Lobatto [64] 2019**	Pituitary cancer	Survival ‡	▪ QoL § [u & g]<br>▪ Endocrine remission §<br>▪ Visual recovery § [cc]<br>▪ Recovery of pituitary function § [dd]	▪ Time to recovery of visual function § [cc]<br>▪ Time to recovery of HRQoL §	▪ Length of hospital stay ‡<br>▪ Nasal morbidity ‡<br>▪ Complications ‡	▪ Persistent disease Recurrences ‡	▪ New pituitary deficiencies ‡<br>▪ Reoperation >30 days and <6 months ‡	Not reported in the study

Author, Year	Condition/ Disease	Survival	Degree of Health or Recovery	Time to recovery & time to return to normal activities	Disutility of care or treatment process	Sustainability of health or recovery	Long-term consequences of therapy	Process Measures
			▪ Degree of resection §	▪ Time to return to work §			▪ Chronic supplementation of pituitary deficits‡<br>▪ Recurrence ‡<br>▪ Persistent hormone excess‡	
**Liang [65] 2015**	Ovarian Cancer	Not reported in the study	Not reported in the study	Not reported in the study	Not reported in the study	Not reported in the study	Not reported in the study	▪ Operative report with documentation of residual disease within 48 h of cytoreduction for women with invasive ovarian, fallopian tube, or peritoneal cancer ‡<br>▪ Complete staging for women with invasive stages I–IIIB ovarian, fallopian tube, or peritoneal cancer who have undergone cytoreduction ‡<br>▪ Intraperitoneal chemotherapy offered within 42 days of optimal cytoreduction to women with invasive stage III ovarian, fallopian tube, or peritoneal cancer ‡<br>▪ Intraperitoneal chemotherapy administered within 42 days of optimal cytoreduction to women with invasive stage III ovarian, fallopian tube, or peritoneal cancer ‡<br>▪ Platin or taxane administered within 42 days following

Author, Year	Condition/ Disease	Survival	Degree of Health or Recovery	Time to recovery & time to return to normal activities	Disutility of care or treatment process	Sustainability of health or recovery	Long-term consequences of therapy	Process Measures
								cytoreduction to women with invasive stages I (grade 3), IC-IV ovarian, fallopian tube, or peritoneal cancer ‡<br>▪ Venous thromboembolism prophylaxis administered within 24 h of cytoreduction to women with invasive ovarian, fallopian tube, or peritoneal cancer ‡<br>▪ Order for prophylactic parenteral antibiotic administration within 1–2 h before cytoreduction for women with invasive ovarian, fallopian tube, or peritoneal cancer ‡<br>▪ Order for prophylactic parenteral antibiotic discontinuation within 24 h after cytoreduction for women with invasive ovarian, fallopian tube, or peritoneal cancer ‡
**Landercasper [66] 2018**	Breast cancer	Not reported in the study	Not reported in the study	Not reported in the study	▪ Surgical site infection and cellulitis after breast and/or axillary surgery ‡<br>▪ Unplanned 30 day reoperation rate after mastectomy ‡	Not reported in the study	Not reported in the study	▪ Preoperative diagnosis of breast cancer ‡<br>▪ Image confirmation of successful excision of image-localized breast lesion ‡

Author, Year	Condition/ Disease	Survival	Degree of Health or Recovery	Time to recovery & time to return to normal activities	Disutility of care or treatment process	Sustainability of health or recovery	Long-term consequences of therapy	Process Measures
								▪ Sentinel lymph node biopsy for invasive breast cancer ‡<br>▪ Surgeon assessment for hereditary cause of breast cancer ‡<br>▪ Specimen orientation for partial mastectomy or excisional breast biopsy ‡<br>▪ Selection of prophylactic antibiotics: first- or second-generation cephalosporin ‡<br>▪ Discontinuation of prophylactic parenteral antibiotics ‡
**Fayanju [67] 2016**	Breast Cancer	▪ Overall survival ‡<br>▪ Disease-free survival‡	▪ Return to normal activities of daily living §<br>▪ Return to work status §	▪ Access to care (≤ 10 days) ‡<br>▪ Treatment delay (>30 days) ‡	▪ Reoperation ‡<br>▪ Treatment-related emergency department visits ‡<br>▪ Readmission ‡<br>▪ Infection requiring antibiotics ‡	▪ Recurrence † [&] ‡	▪ Functional assessment of cancer therapy § [ee] (appearance satisfaction, patient-reported lymphedema, fatigue, physical intimacy, pain, physical well-being, social well-being, fertility preservation, cognitive dysfunction, neuropathy, osteoporosis)	Not reported in the study
**Cramer-van der Welle [68] 2021**	Lung cancer	▪ Overall survival after Diagnosis ‡<br>▪ Overall mortality 1 and 2 years after diagnosis ‡	▪ QoL § [q & ff]<br>▪ Treatment result after resection: Resection margins ‡	Not reported in the study	▪ Complications after resection ‡<br>▪ Side effects after radiotherapy or chemotherapy ‡	Not reported in the study	Not reported in the study	Not reported in the study

Author, Year	Condition/ Disease	Survival	Degree of Health or Recovery	Time to recovery & time to return to normal activities	Disutility of care or treatment process	Sustainability of health or recovery	Long-term consequences of therapy	Process Measures
			▪ Treatment result after resection: Rethoracotomy‡					
**Morgans [69] 2015**	Prostate Cancer	▪ Cause-specific survival † ▪ Overall survival †	▪ QoL § [q & aa] ▪ Performance status ‡ [gg] ▪ Need for pain medication §	Not reported in the study	▪ Major systemic therapy complications ‡	▪ Procedures needed for local progression ‡ ▪ Symptomatic skeletal event ‡ ▪ Development of metastasis ‡ ▪ Development of Castration-Resistant Disease ‡	Not reported in the study	Not reported in the study
**Mak [70] 2016**	Lung cancer	▪ Overall survival † ▪ Treatment-related mortality ‡ ▪ Cause of death †	▪ QoL § [q & ff] ▪ Performance status ‡ [gg] ▪ Quality of death † [&] ‡	▪ Time from diagnosis to treatment ‡	▪ Major surgical complications ‡ ▪ Major radiation complications ‡ ▪ Major systemic therapy complications ‡	Not reported in the study	Not reported in the study	Not reported in the study
**Lagendijk [71] 2018**	Breast cancer surgeries	Not reported in the study	▪ QoL § [u, q, v & w]	Not reported in the study	Not reported in the study	Not reported in the study	Not reported in the study	Not reported in the study
**Javid [72] 2017**	Breast Cancer	Not reported in the study	▪ QoL (Physical function; Treatment decision-making; Body image; Emotional well-being; Sexual function) §	Not reported in the study	Not reported in the study	Not reported in the study	Not reported in the study	Not reported in the study
**Escudero-Vilaplana [73] 2020**	Lung cancer	▪ Overall survival† ▪ Cause of death ‡	▪ QoL § [u & hh] ▪ Performance status ‡ [gg] ▪ Quality of death † [&] ‡ [&] § ▪ Patient productivity loss (Sick leave or disability) §	▪ Time from diagnosis ‡ ▪ Time from diagnosis to Treatment ‡	▪ Major surgical complications ‡ ▪ Major systemic therapy or/and radiotherapy complications ‡	Not reported in the study	Not reported in the study	Not reported in the study
**Ong [74] 2017**	Breast cancer	▪ Overall survival† ▪ Death attributed to breast cancer† ▪ Recurrence-free survival (local,	▪ QoL § [q, s, v, w, x]	Not reported in the study	▪ Reoperations owing to involved margins ‡ [and/or] § ▪ Severity of acute complications based on the	Not reported in the study	Not reported in the study	Not reported in the study

Author, Year	Condition/ Disease	Survival	Degree of Health or Recovery	Time to recovery & time to return to normal activities	Disutility of care or treatment process	Sustainability of health or recovery	Long-term consequences of therapy	Process Measures
		regional, or distant)‡			Clavien-Dindo and CTCAE ‡ and/or §<br>▪ Name of acute complication ‡			

Abbreviations: ACE, Angiotensin-Converting Enzyme; ARB, Angiotensin Receptor Blockers; AVD, Aortic Valve Disease; AVR, Aortic Valve Replacement; CABG, Coronary Artery Bypass Grafting; CAD, Coronary Artery Disease; CT, Conservative Treatment; CTCAE, US National Cancer Institute Common Terminology Criteria for Adverse Events; cT Stage, Clinical T Stage; HRQoL, Health-Related Quality of Life; PCI, Percutaneous Coronary Intervention; QoL, Quality of Life; SAVR, Surgical Aortic Valve Replacement; TAVI, Transcatheter Aortic Valve Implantation; TAVR, Transcatheter Aortic Valve Replacement; VTE, Venous Thromboembolism.

Data source: † Administrative; ‡ Clinician-Reported; § Patient-Reported.

Patient-Reported Outcome Measures Instruments: [a] European Quality of Life (EQ-5D-3L); [b] Patient-Reported Outcomes Measurement Information System (PROMIS Global-10); [c] Short Form–12 (SF-12); [d] Veterans RAND -12 (VR-12); [e] Beliefs about Medicine Questionnaire (BMQ); [f] Hill-Bone Medication Adherence Scale; [g] Short Form–36 (SF-36); [h] simplified modified Rankin Scale questionnaire; [i] Patient Health Questionnaire–4 (PHQ-4); [j] Kansas City Cardiomyopathy Questionnaire (KCCQ); [k] Quality of Life Index Cardiac Version (QLI); [l] Quality of Life after Myocardial Infarction (QLMI); [m] Seattle Angina Questionnaire (SAQ–7); [n] Rose Dyspnea Scale; [o] Patient Health Questionnaire–2 (PHQ-2); [p] Physical Function Short Form-4a; [q] European Organization for Research and Treatment of Cancer–Core 30 (EORTC–C30); [r] European Organization for Research and Treatment of Cancer–Colorectal cancer-29 (EORTC–CR29); [s] European Organization for Research and Treatment of Cancer – Colorectal Liver Metastasis-21 (EORTC–LMC21); [t] Memorial Sloan-Kettering Cancer Center-Bowel Function Instrument (MSKCC-BFI); [u] European Quality of Life (EQ-5D-5L); [v] European Organization for Research and Treatment of Cancer–Breast 23 (EORTC–BR23); [w] Breast Questionnaire (Breast Q); [x] Functional Assessment of Cancer Therapy - Endocrine Symptoms (FACT–ES); [y] Reproductive Concerns Scale (RCS); [z] Care-related Quality of Life (CarerQol-7D); [aa] Expanded Prostate Cancer Index -26 (EPIC-26); [bb] Service Satisfaction Scale (SSS-CC); [cc] Visual Function Questionnaire (VFQ–25); [dd] Leiden Bother and Needs Questionnaire for patients with pituitary disease (LBNQ-Pituitary); [ee] Functional Assessment of Cancer Therapy – Breast (FACT-B); [ff] European Organization for Research and Treatment of Cancer – Lung Cancer-13 (EORTC–LC 13); [gg] Eastern Cooperative Oncology Group Scale of Performance Status (ECOG Scale of Performance Status); [hh] Specific questionnaire Lung Cancer Symptom Scale (LCSS) in Spain.
